# Holey two-dimensional transition metal oxide nanosheets for efficient energy storage

**DOI:** 10.1038/ncomms15139

**Published:** 2017-04-26

**Authors:** Lele Peng, Pan Xiong, Lu Ma, Yifei Yuan, Yue Zhu, Dahong Chen, Xiangyi Luo, Jun Lu, Khalil Amine, Guihua Yu

**Affiliations:** 1 Materials Science and Engineering Program and Department of Mechanical Engineering, The University of Texas at Austin, Austin, Texas 78712, USA; 2 Chemical Sciences and Engineering Division, Argonne National Laboratory, Argonne, Illinois 60439, USA

## Abstract

Transition metal oxide nanomaterials are promising electrodes for alkali-ion batteries owing to their distinct reaction mechanism, abundant active sites and shortened ion diffusion distance. However, detailed conversion reaction processes in terms of the oxidation state evolution and chemical/mechanical stability of the electrodes are still poorly understood. Herein we explore a general synthetic strategy for versatile synthesis of various holey transition metal oxide nanosheets with adjustable hole sizes that enable greatly enhanced alkali-ion storage properties. We employ *in-situ* transmission electron microscopy and operando X-ray absorption structures to study the mechanical properties, morphology evolution and oxidation state changes during electrochemical processes. We find that these holey oxide nanosheets exhibit strong mechanical stability inherited from graphene oxide, displaying minimal structural changes during lithiation/delithiation processes. These holey oxide nanosheets represent a promising material platform for *in-situ* probing the electrochemical processes, and could open up opportunities in many energy storage and conversion systems.

## Abstract (web summary)

As alkali-ion battery anodes, metal oxide nanomaterials suffer from severe structural degradation after charging/discharging cycling. Here the authors develop two-dimensional holey nanosheet anodes which display minimal structural changes during electrochemical operation.

Two-dimensional (2D) nanocrystals offer exciting opportunities for both fundamental studies and many technological applications due to their unique and fascinating properties^1^,^2^,^3^. This has been highlighted over the past decade by 2D graphene and transition metal dichacolgenides, which exhibit exceptional chemical/physical properties that are absent in their bulk counterparts and other dimensional nanostructures^4^,^5^,^6^,^7^,^8^. Mixed transition metal oxide (MTMO) nanomaterials have been widely studied as attractive candidates for electrocatalysis, photocatalysis, energy storage and conversion technologies, owing to their mixed valence states and rich redox reactions^9^,^10^,^11^,^12^,^13^. Inspired by the success of 2D nanomaterials as mentioned above, it is of great interest to fabricate the MTMO materials into 2D nanostructures, which are expected to exhibit greatly improved properties in many energy-related applications, especially alkali ion storage. However, up to now, MTMOs are mainly studied in the form of 0D nanoparticles^14^,^15^, 1D nanotubes or nanowires^16^,^17^, and 3D nanoclusters or microspheres^18^. In contrast, there are only a few reports studying the 2D nanostructured MTMOs, especially those with confined thickness^19^,^20^,^21^. MTMOs are intrinsically non-layered materials, which cannot be mechanically or chemically exfoliated to 2D nanostructures via the general top-down exfoliation methods^22^,^23^,^24^. Therefore, a general and facile bottom-up strategy for controlled synthesis of 2D MTMO nanostructures is highly needed.

Although 2D nanomaterials generally exhibit improved capacity, rate capability and cycling stability for Li ion storage due to the abundant active sites for electrochemical reaction and the shortened Li ion diffusion distance, they still suffer from several disadvantages. 2D nanomaterials with high surface areas may consume more electrolytes for the formation of SEI, and cause more unwanted side reactions to deteriorate the battery performance^2^,^25^,^26^. 2D nanomaterials suffer from decrease of active surfaces for ion transport and storage due to the irreversible restacking of individual 2D nanosheets^27^,^28^. Moreover 2D TMO nanomaterials also suffer from severe morphology changes and structural degradation, especially in the first discharge cycle, due to the reduction of the metal cation to M(0) (refs 29, 30, 31). For example, Tarascon *et al*. reported cobalt oxide (CoO) nanoparticles as a good anode material with high Li^+^ storage capacity and capacity retention. The authors proved that when CoO nanoparticles were fully reduced by Li, the overall shape of the starting material can be preserved because the disintegrated metallic nanoparticles would be dispersed in a lithia (Li_2_O) matrix, with the Li_2_O+nanoparticles being surrounded by a solid electrolyte interface (SEI)^32^. Over the past decades, there is a great amount of research on simple and mixed transition metal oxides as lithium ion battery and sodium ion battery anodes^33^,^34^,^35^,^36^, but detailed conversion reaction process in terms of the oxidation state changes (chemical stability) and morphology evolution (mechanical stability) are still relatively poorly understood. Meanwhile, the chemical/mechanical stability of these metal oxides and the detailed understanding of their oxidation state changes during electrochemical processes are crucial for further optimization of electrochemical performance.

Here we report a general two-step strategy for controlled synthesis of holey 2D TMO nanosheets with tunable pore sizes using graphene oxides as a sacrificial template (Fig. 1). This approach has been demonstrated to synthesize various 2D holey TMO nanosheets, including simple oxides such as Fe_2_O_3_, Co_3_O_4_, Mn_2_O_3_, and mixed oxides such as ZnMn_2_O_4_ (ZMO), ZnCo_2_O_4_ (ZCO), NiCo_2_O_4_ (NCO) and CoFe_2_O_4_ (CFO). As a result, 2D holey TMO nanosheets exhibit much improved rate capability and cycling stability for both lithium and sodium ion storage, due to the increased surface areas and interfaces, and facile interfacial transport and shortened diffusion paths. What's more, the operando X-ray diffraction (XRD) and X-ray absorption structures (XAS) are employed to investigate the charge storage mechanism in the conversion reaction involving 2D holey ZnMn_2_O_4_ (ZMO) nanosheets and to understand the oxidation state changes of Zn and Mn elements. We also use *in-situ* transmission electron microscopy (TEM) to follow the morphology evolution of the 2D holey ZMO nanosheets during lithiation/delithiation and pressing process in real time. Operando XRD and XAS results show that holey ZMO nanosheets deliver high capacity due to the formation of ZnLi alloy as well as the reversible transformation between Mn^2+^ and Mn^3+^. *In-situ* TEM characterizations show that 2D holey ZMO nanosheets composed of chemically interconnected metal oxide nanoparticles inherit the strong mechanical properties from graphene oxide, maintaining the holey morphology and displaying minimal structural changes during the lithiation/delithiation processes and under press states. The electrochemical results, in combination of the *in-situ* TEM, operando XRD and XAS studies, show that these 2D holey nanostructured TMO materials are a promising material platform for both fundamental understanding of the electrode stability during lithiation/delithiation, and for improving electrochemical performance because of the synergistic effects of the inherently good chemical/mechanical stability and the enhanced charge transport properties.

## Results

### Synthesis and characterization of 2D holey MTMO nanosheets

**Figure 1:**
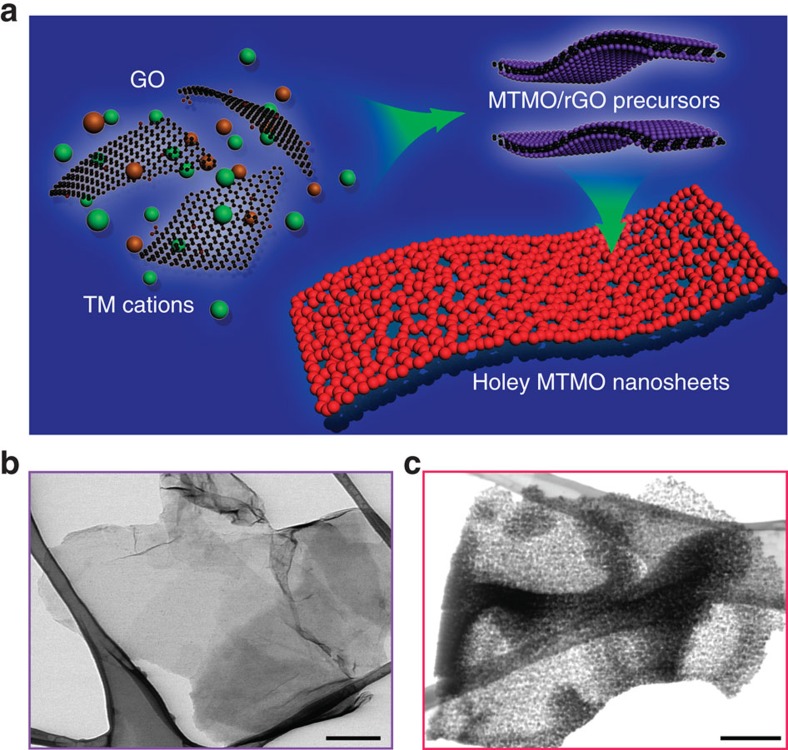
Schematic illustration of the synthesis process of 2D holey TMO nanosheets. (**a**) Schematic showing the general strategy to synthesize 2D holey TMO nanosheets. Two transition metal (TM) cations are mixed with graphene oxide (GO) and then anchored on surfaces of reduced graphene oxide (rGO) templates during solution-phase reaction. 2D holey MTMO nanosheets composed of interconnected MTMO nanocrystals are formed after removing rGO templates during post-calcination. (**b**) STEM image of ZMO precursor/rGO shows sheets-like morphology. (**c**) STEM image of 2D holey ZMO nanosheets shows holey nanosheets composed of interconnected ZMO nanocrystals. Scale bars, 200 nm (**b**,**c**).

The key concept for general synthesis of 2D holy TMO nanosheets is illustrated in Fig. 1a. In brief, GO was first employed as a template to grow various TMO precursors on its surfaces, followed by post-thermal treatment to transform TMO precursors to 2D holey TMO nanosheets owing to the synergistic effects of chemical interconnection of TMO nanoparticles and controlled decomposition of GOs. In a typical experiment, TMO precursor/rGO composites were firstly prepared via solution-phase reaction between transition metal ions and GO, which was partially reduced to reduced graphene oxide (rGO)^37^,^38^. The resulting TMO precursor/rGO were then annealed to induce pyrolysis of rGO templates and formation of crystallized TMO nanoparticles, which interconnected chemically with each other to form the 2D holey nanosheets. Taking 2D holey ZMO nanosheets as an example, GO was firstly dispersed in ethylene glycol solution by ultrasonication. Afterwards, Zn(CH_3_COO)_2_ and Mn(CH_3_COO)_2_ were added into GO suspensions. Then stable and homogenous suspensions were obtained by stirring to ensure the complete adsorption of Zn^2+^ and Mn^2+^ cations onto the surfaces of GO. After refluxing, the black ZMO precursors/rGO precipitates were washed and collected by centrifugation. Low magnification scanning transmission electron microscope (STEM) image (Fig. 1b) shows a typical sheet-like morphology of the ZMO precursors/rGO, indicating that rGO served as a general template for supporting the ZMO precursors. A close-up STEM image (Supplementary Fig. 1a) indicates that ZMO precursors were well distributed on the rGO sheets. X-ray powder diffraction pattern (Supplementary Fig. 1b) suggests that the as-synthesized ZMO precursors were amorphous. No diffraction peaks of rGO were observed indicating well exfoliation of rGO nanosheets. The STEM (Supplementary Fig. 1c) and corresponding elemental mappings (Supplementary Fig. 1d) show the uniform distributions of Zn, Mn, O, C elementals, further confirming the ZMO precursors were uniformly anchored on the surfaces of rGO nanosheets.

**Figure 2:**
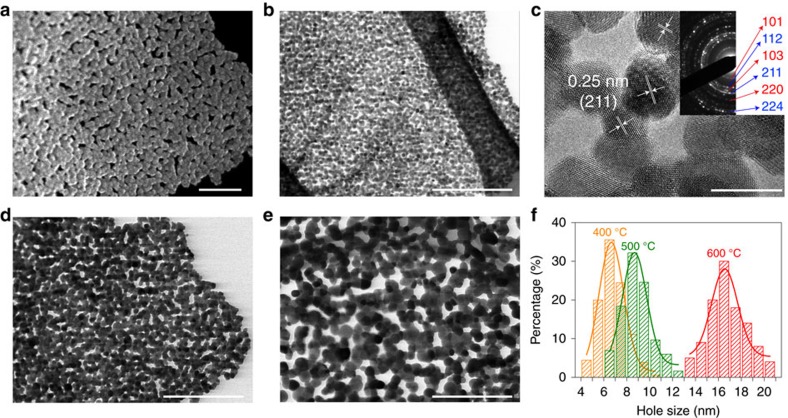
Electron microscopic images of 2D holey ZMO nanosheets. SEM image (**a**), STEM image (**b**) and high-magnification TEM image (**c**) of 2D holey ZMO nanosheets prepared at post-calcination temperature of 400 °C. Corresponding SAED pattern of 2D holey ZMO nanosheets (inset of **c**). STEM image of 2D holey ZMO nanosheets prepared at different post-calcination temperature: (**d**) 500 °C and (**e**) 600 °C. Corresponding hole size distribution (**f**) obtained by statistics analysis of the STEM images shown in **b**,**d**,**e**. Scale bars, 100 nm (**a**), 200 nm (**b**,**d**,**e**), 10 nm (**c**).

Post-calcination of the as-prepared ZMO precursors/rGO induced the transformation of amorphous precursors into crystalline ZMO (Supplementary Fig. 2a) nanosheets without altering their 2D morphology, and simultaneous decomposition of rGO (Supplementary Fig. 2b). The typical STEM images (Fig. 1c and Supplementary Fig. 2c) of the samples after calcination confirm that 2D nanosheet structures had been reserved in the final products. However, the nanosheets had been transformed from a dense structure with smooth surfaces (Fig. 1b and Supplementary Fig. 1a) into a highly holey nanosheet (Fig. 2a,b). The as-synthesized holey nanosheets showed good uniformity in lateral size (∼500 nm) and thickness (∼20 nm) (Supplementary Figs 3 and 4). HRTEM (Fig. 2c) image reveals the 2D holey nanosheets consist of interconnected nanocrystallites of ∼8 nm in size. Moreover, the crossed two fringes in the overlapped domains (Supplementary Fig. 5) reveal that the ZMO nanoparticles indeed are chemically connected with each other. The clear lattice fringes of ∼0.25 nm (Fig. 2c) correspond well to the (211) facet of the spinel ZMO. The diffused concentric rings shown in selected area electron diffraction (SAED) pattern (inset of Fig. 2c) indicate the polycrystalline structure. The diameters of the diffraction rings are indexed to spinel ZMO in agreement with the XRD analysis (Supplementary Fig. 2a). The STEM (Supplementary Fig. 2c) and corresponding elemental mapping (Supplementary Fig. 2d) display the uniform distribution of Zn, Mn, O elements with rare C element, which further demonstrates that the 2D holey nanosheets are composed of the ZMO nanoparticles.

GO is playing an important role in the formation of 2D holey ZMO nanosheets. First, GO is a 2D template with sufficient oxygen-contained groups, which ensures the growth of ZMO precursors on its surfaces. This is essential for the formation of 2D nanostructure. Second, unlike the conventional templating process where little interactions exist between the precursors and templates, the ZMO precursors were anchored covalently on rGOs through residual functional group such as carboxyl, hydroxyl and epoxy groups (Supplementary Fig. 6)^39^,^40^. Owing to the intimate interaction between ZMO and rGO, the ZMO nanoparticles partially agglomerated and chemically linked with each other to form the holey nanosheets during the thermal treatment, as identified by the HRTEM of ZMO nanoparticles. Third, rGO template is highly flexible, which may contribute to maintaining the structure stability of 2D holey nanosheets during calcination. Owing to the chemically integration between ZMO precursors and GO, the as-synthesized ZMO nanoparticles inherited the strong mechanical properties from the GO nanosheets. Free ZMO was synthesized via the same method without any GO added as a control experiment. Only an aggregated flower-like structure assembled by spinel ZMO discs was obtained (Supplementary Fig. 7a,b). No holey nanosheet structures were formed (Supplementary Fig. 7c,d).

In many electrochemical energy devices, it is highly desirable to control nanomaterials with tunable pore/hole sizes, since the opening of holes or pores may have a significant effect on their electrochemical performances^41^,^42^. This is particularly true for 2D nanomaterials as when processed as electrode materials, restacking of nanosheets often leads to substantially reduced active surfaces for electrochemical reactions and suppressed mass transport. In our work, the hole size of 2D holey ZMO nanosheets prepared by such strategy can be adjustable by readily tuning the annealing temperature during post-calcination process. Figure 2b,d,e shows STEM images of 2D holey ZMO nanosheets prepared by calcination at 400, 500 and 600 °C, respectively. The 2D holey nanosheets structure was well maintained at all temperatures, while the ZMO particles grew bigger and showed more agglomerated as the temperature increases. From Fig. 2f it is evident that the hole size of 2D holey ZMO nanosheets increased with the increasing calcination temperature. The hole sizes of 2D holey ZMO nanosheets obtained at different temperatures were further tested and verified by the BET characterizations (Supplementary Fig. 8). The BET results gave the average hole sizes of 5∼10, 7∼17 and ∼20 nm for the samples obtained at 400, 500 and 600 °C, respectively, which were consistent with the values obtained from the STEM images.

More importantly, such strategy is versatile and can be extended to prepare other 2D holey MTMO nanosheets. Supplementary Fig. 9a,d,g shows scanning electron microscope (SEM) images of 2D holey ZnCo_2_O_4_ (ZCO), NiCo_2_O_4_ (NCO) and CoFe_2_O_4_ (CFO) nanosheets prepared by the same approach, respectively. STEM images of 2D holey ZnCo_2_O_4_ (ZCO), NiCo_2_O_4_ (NCO) and CoFe_2_O_4_ (CFO) nanosheets were shown in Supplementary Fig. 9b,e,h, respectively. In comparison with the 2D ZMO nanosheets shown in Fig. 2a,b, similar 2D holey structures were clearly observed in all these cases. HRTEM (Supplementary Fig. 9c,f,i) images reveal these 2D holey nanosheets all consist of chemically interconnected MTMO nanocrystallites. The lattice fringes (Supplementary Fig. 9c,f,i) and SAED patterns (inset of Supplementary Fig. 9c,f,i) confirm that the 2D ZCO, NCO and CFO nanosheets were composed of spinel ZCO, NCO and CFO, respectively, corresponding well to XRD results (Supplementary Fig. 10). In addition to MTMOs shown above, this strategy can be also applied to the synthesis of simple TMOs, such as Fe_2_O_3_, Co_3_O_4_, and Mn_2_O_3_. The STEM images displaying the 2D holey morphology of simple TMOs were shown in Supplementary Fig. 11.

### 2D holey MTMO nanosheets as anodes for lithium-ion battery

**Figure 3:**
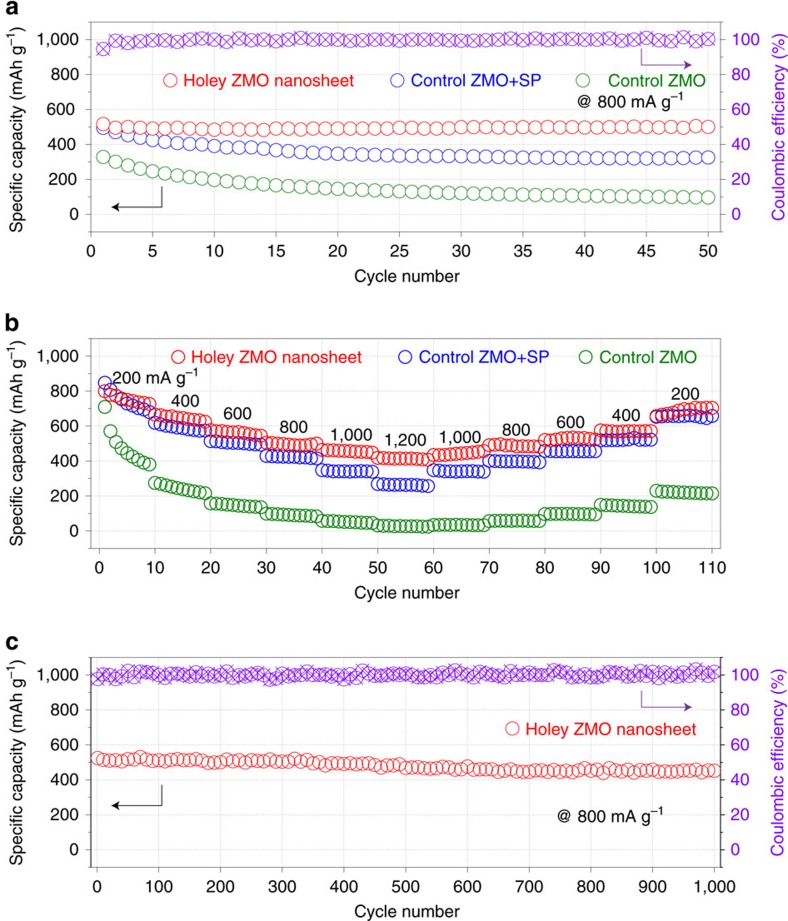
2D holey ZMO nanosheets as high-performance anodes for lithium-ion batteries. (**a**) Cycling performance of 2D holey ZMO nanosheets, control ZMO+SP and control ZMO at current density of 800 mA g^−1^ for 50 cycles. Coulombic efficiency of 2D holey ZMO nanosheets at current density of 800 mA g^−1^ for 50 cycles. (**b**) Rate performance of 2D holey ZMO nanosheets, control ZMO+SP and control ZMO at different current densities from 200 to 1,200 mA g^−1^. (**c**) Long-term cycling stability and Coulombic efficiency of 2D holey ZMO nanosheet at current density of 800 mA g^−1^ for over 1,000 cycles.

Prompted by their unique morphology, these 2D holey MTMO nanosheets are expected to show promising applications in electrochemical energy storage devices. For example, when used as anodes for lithium-ion batteries, these 2D holey nanosheets may afford large surface areas and enable facile transport of the electrolyte, resulting in rapid charge transfer due to the shortened diffusion paths^18^. Supplementary Fig. 12a shows the charge and discharge curves of 2D holey ZMO nanosheets for the first two cycles in the range of 0.01 and 3.0 V. The voltage profile of the first Li^+^ charge comprises mainly two regions, a large plateau at 0.5 V associated with the irreversible reaction between Li^+^ and ZMO, which is followed by a slope till 0.01 V. The Li^+^ discharge curve shows no large plateau but only a slope due to oxidation reactions of Mn^0^ and Zn^0^ to Mn^2+^ and Zn^2+^. The following charge and discharge slopes reflect the reversible reactions between Mn^0^, Zn^0^ and Mn^2+^, Zn^2+^ and Zn–Li alloying/de-alloying reactions. The initial capacity loss is attributed to the formation of SEI^43^. After several conditioning cycles, the Coulombic efficiency increased to higher than 98% (Fig. 3a), indicating good reversibility of the above conversion reactions. After initial two cycles for activation, a stable specific capacity of ca. 500 mA h g^−1^ (the mass of active materials were used to calculate specific capacities) can be observed at a current density of 800 mA g^−1^ for 50 cycles. The control ZMO+SP and control ZMO samples (synthesized without GO template) that cycled at the same conditions (Fig. 3a) only deliver specific capacities of ca. 320 and ca. 100 mA h g^−1^, respectively.

The rate capability of the as-prepared 2D holey ZMO nanosheets is compared with control ZMO+SP and control ZMO as well (Fig. 3b). For the first few cycles at a low current density of 200 mA g^−1^, the 2D holey ZMO nanosheets show an average specific capacity of ca. 770 mA h g^−1^ (Supplementary Fig. 12b). A high capacity of∼430 mA h g^−1^ (Supplementary Fig. 12b) with a ∼56% capacity retention can be achieved at a high current density of 1,200 mA g^−1^. An average specific capacity of∼710 mA h g^−1^ at 200 mA h g^−1^ is retained after 110 cycles of charge and discharge at various current densities. However, for control ZMO+SP and control ZMO, only ∼32% and ∼6% capacity retention are obtained respectively, as current density increased from 200 to 1,200 mA h g^−1^. The superior rate performance is also confirmed in the 2D holey ZMO sample prepared at different temperatures (Supplementary Figs 13 and 14). The samples treated at 400 and 500 °C show the comparable electrochemical performance. While a prominent decrease of rate performance is observed when the calcination temperature increased to 600 °C (Supplementary Fig. 14). The more aggregated structure and bigger particle size may be attributed to the decreased performance. Furthermore, cycling stability measurement of 2D holey ZMO nanosheets is given in Fig. 3c. After five cycles of electrochemical activation at 100 mA g^−1^, a stable specific capacity of ca. 480 mA h g^−1^ can be retained after 1,000 cycles at the current density of 800 mA g^−1^. And the average Coulombic efficiency from the 1st to 1,000th cycle is 99.8%. The excellent Coulombic efficiency can be attributed to the electrochemical activation process, which facilitates the formation of SEI layers^44^,^45^. The 2D holey CFO, ZCO and NCO nanosheets can also serve as anodes for lithium storage. The CV curves of the holey ZCO, holey CFO and holey NCO nanosheets at scanning rates of 0.1 mV s^−1^ are showed in Supplementary Fig. 15. Long-term cycling stabilities of these 2D holey nanosheets are shown in Supplementary Fig. 16a–c, respectively. All these 2D holey nanosheets exhibit high cycling stability revealing that this strategy is a general route for synthesis of 2D holey nanomaterials with exceptional lithium storage ability. The cycling stability and rate capability of these 2D holey MTMO nanosheets are compared with the reported MTMO-based anodes (Supplementary Table 1). The 2D holey MTMO nanosheets exhibit superior performances. To the best of our knowledge, this is the first time that an MTMO-based anode with such a long cycling stability has been reported.

The high rate capability, excellent cyclic stability and Coulombic efficiency of 2D holey ZMO nanosheets can be ascribed to the unique features of 2D holey nanostructures with favourable properties. Firstly, the 2D nanosheets with large surface areas and short diffusion length facilitate effective lithium ion transport. Secondly, the interconnected holes on the surfaces of 2D nanosheets facilitate the liquid electrolyte diffusion into the bulk of the electrode materials and greatly reduce the Li^+^ ion diffusion length (Supplementary Fig. 17)^42^,^46^. Even without adding conductive carbon in the electrodes, electrons could be easily transported continuously along well-interconnected nanocrystals. This is particularly helpful for the battery performance at high rates. Finally, these interconnected holes can help accommodate the volume changes during lithiation/delithiation, thus enhancing the cycling stability and Coulombic efficiency^47^. As shown in Supplementary Fig. 18, 2D holey ZMO nanosheets could still preserve the structure integrity and holey structure after 100 cycles. Such 2D holey nanostructures might endow these materials with many intriguing applications including supercapacitors, catalysis, electrochemical sensors, and so on.

### 2D holey MTMO nanosheets as anodes for sodium-ion battery

**Figure 4:**
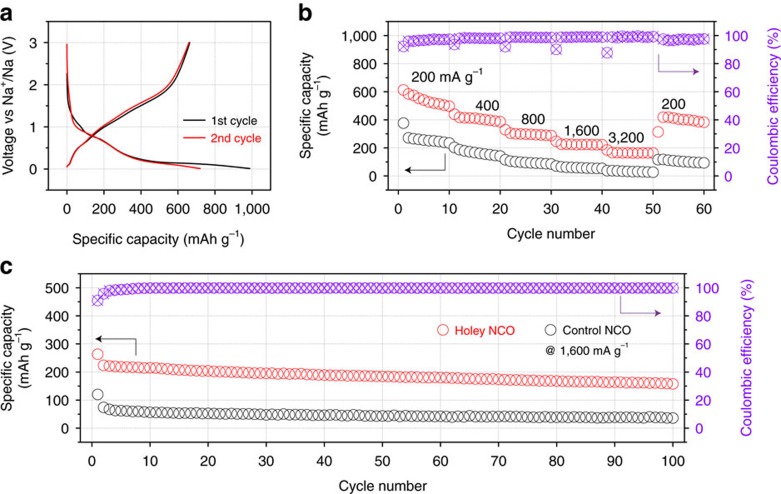
2D holey NCO nanosheets as anodes for sodium-ion batteries. (**a**) Charge/discharge profiles of 2D holey NCO nanosheets for the first and second cycles at a current density of 100 mA g^−1^. (**b**) Rate performances of 2D holey NCO nanosheets (red circle) and control NCO nanosheets (black circle) from 200 to 3,200 mA g^−1^. (**c**) Long-term cycling stability and Coulombic efficiency of 2D holey NCO nanosheet and control NCO nanosheet at current density of 1,600 mA g^−1^ for 100 cycles.

As discussed above, 2D holey MTMO nanosheets possess both 2D nanostructure and tunable porosity, which may contribute to greatly improved electrochemical performance compared to conventional nanosheets electrodes with smooth surfaces. To demonstrate the advantageous features for electrochemical energy storage, 2D holey MTMO nanosheets are further explored as anodes for sodium-ion batteries. The charge/discharge curves of 2D holey NCO nanosheets for the first and second cycles at 100 mA g^−1^ are shown in Fig. 4a. The CV curves of 2D holey NCO nanosheets for first three cycles at 0.1 mV s^−1^ are shown in Supplementary Fig. 19. In the first discharge cycle, a sloping curve is shown in the potential range of 0.2∼1.2 V and a flat plateau can be observed at 0.2 V. According to the literature^48^,^49^, reduction of NCO to metallic Co and Ni nanoparticles and formation of Na_2_O (NiCo_2_O_4_+8Na^+^+8e^−^→Ni+2Co+4Na_2_O) occurs at the plateau at 0.2 V. The small sloping plateau at 0.7 V corresponds to the formation of an SEI film. An initial discharge capacity of ∼980 mA h g^−1^ and a reversible capacity is ∼670 mA h g^−1^ with a Coulomb efficiency of∼68.4% can be achieved. A Coulombic efficiency of around 96% is reached in the following cycles, indicating that the charge/discharge curves are stabilized gradually. Figure 4b and Supplementary Fig. 20 compare the rate performance of the 2D holey NCO nanosheets and control NCO nanoplates without porosity (Supplementary Fig. 21) for SIBs. At current densities of 200, 400, 800, 1,600 and 3,200 mA g^−1^, the 2D holey NCO nanosheets deliver the capacities of ca. 550, 420, 300, 230 and 170 mA h g^−1^, respectively. The holey naosheets electrodes still deliver a reversible capacity of 420 mA h g^−1^ when the current density is reverted gradually to 200 mA g^−1^. While the control NCO nanosheets without porosity only deliver a low capacity of ∼60 mA h g^−1^ at the current density of 1,600 mA g^−1^, and can be hardly charged/discharged at higher current density. Figure 4c shows the long-term cycling stability and Coulombic efficiency of 2D holey NCO nanosheets and control NCO nanosheets at current density of 1,600 mA g^−1^ for 100 cycles. A stable specific capacity of ca. 160 mA h g^−1^ was observed after 100 cycles for the 2D holey NCO nanosheet anodes. For comparison, the control anodes, NCO nanosheet without porous structure, showed the specific capacities of ca. 36 mA h g^−1^ after 100 cycles. These high rate capability and excellent cyclic stability can be attributed to the unique 2D holey structure of the NCO nanosheets. The unique 2D holey structure will enable facile diffusion of liquid electrolyte into the bulk of the electrode materials and greatly reduce the Li^+^ ion diffusion length, thus improving the rate capability. Also, these interconnected holes can also maintain the holey framework of the nanosheets and accommodate volume change during charging/discharging, thus improving the cycling stability and Coulombic efficiency.

### *In-situ* TEM studies of 2D holey MTMO nanosheet anodes

**Figure 5:**
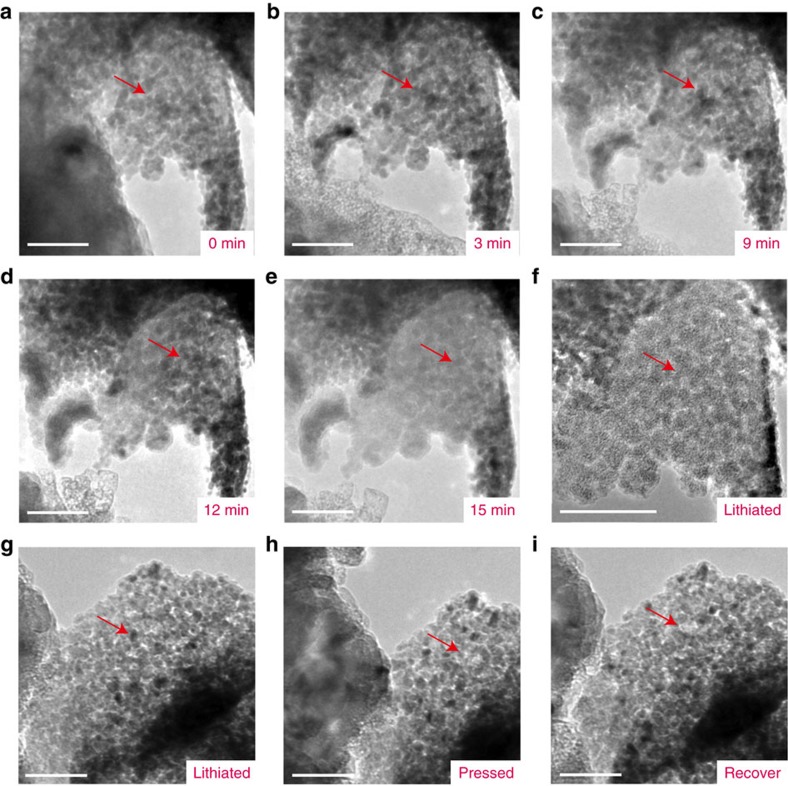
*In-situ* TEM studies of 2D holey ZMO nanosheets as anodes. (**a**–**f**) TEM images of the 2D holey nanosheets at different lithiation stages/times. (**g**–**i**) TEM images of the 2D holey nanosheets under press states. Scale bars, 100 nm. Red arrow points out the holey structures of the 2D ZMO nanosheets.

The high storage capacity for Li/Na, high rate capability and cycling stability of 2D holey nanosheet samples can be ascribed to the 2D interconnected porous framework that provides large active surface areas, bicontinuous Li^+^/Na^+^ and electron pathways and excellent structural stability inherited from the graphene oxide. To further understand the structural stability of 2D holey nanosheets, structural evolution during the lithiation process is carried out by *in-situ* TEM imaging on ZMO nanosheet, as a model material. The first-cycle lithiation process of 2D holey ZMO nanosheets is examined, as shown in Fig. 5. As discussed before, ZMO is fully reduced by Li into Zn and Mn metallic nanoparticles which are dispersed in a lithia (Li_2_O) matrix, with the Li_2_O+nanoparticles being surrounded by an SEI^33^. These results can be further identified by SAED of the fresh and lithiated samples, as shown in Supplementary Fig. 22. The SAED of fresh samples can be indexed to the spinel ZMO structures in agreement with the XRD analysis. This result is different from that of the lithiated samples, which can be indexed to the Zn and Mn metallic phases (Supplementary Fig. 22b). It should be noted that the *in-situ* TEM characterizations during the first lithiation process proved the structural uniqueness and inherently strong mechanical stability of the 2D holey ZMO nanosheets. During the first lithiation (Fig. 5a–f), 2D holey ZMO nanosheets maintain the holey morphology in different lithiation stages/times, for example 3, 9, 12 and 15 min. After full lithiation, the overall shape of the starting material can be preserved in 2D geometry, and the overall morphology remains as holey/porous structures because the disintegrated metallic nanoparticles would be dispersed in a lithia (Li_2_O) matrix. The lithiated ZMO nanosheets can remain its overall morphology and porous nature when the ZMO nanosheet is under press during *in-situ* stress tests, as shown in Fig. 5g–i. These results are consistent with the aforementioned improved electrochemical characteristics for Li/Na ion storage. The 2D holey MTMO nanosheets can maintain the holey framework and accommodate volume change during charging/discharging, thus improving the cycling stability and Coulombic efficiency.

### Operando XAS studies on 2D holey MTMO nanosheets

**Figure 6:**
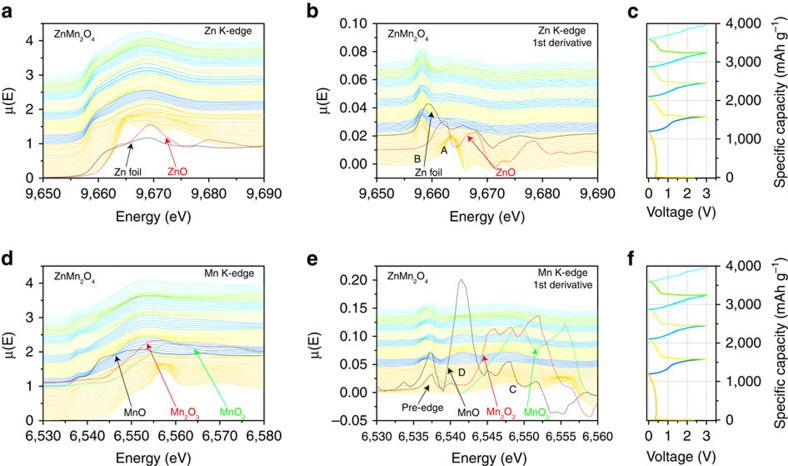
Operando XAS studies on 2D holey MTMO nanosheet. (**a**,**b**) Scan at the Zn K-edge and the corresponding first derivative curves during the charge/discharge process. (**c**) The charge/discharge curves of the 2D holey ZMO nanosheets at 50 mA g^−1^. (**d**,**e**) Scan at the Mn K-edge and the corresponding first derivative curves during the charge/discharge process. (**f**) The charge/discharge curves of the 2D holey ZMO nanosheets at 50 mA g^−1^.

As the spinel structure of MTMO is not recovered after the first cycle, operando XAS was further applied as a powerful method to provide detailed information about the oxidation states and the local environment changes of atoms on a short-range scale. The XAS scans of the Zn K-edges and Mn K-edge and their corresponding first derivative curves during the charge/discharge processes are shown in Fig. 6. The edge position of the XAS spectra is related to the oxidation state. Initially, the edge energy of the Zn K-edge was 9,664 eV, which is a little higher than that of Zn^2+^, indicating that the oxidation state of Zn is a little higher than 2+ (peak A in the first derivative plot is at a higher energy than ZnO standard) (Fig. 6a,b). This is probably due to the formation of the intermediate NaCl-type structure in which the Zn^2+^ has tetrahedral coordination^50^,^51^. During formation of the intermediate NaCl-type structure, the intensity of the 1*s*→4*p* transition increases for the Zn K-edge. For the discharge process, the intensity of this spectral feature progressively decreases, which can be explained by the loss of *p* character of the 1*s*→4*p* transition as a consequence of the formation of metallic Zn nanoparticles, which occupies the 4*p* orbital. Metallic Zn nanoparticles further form the ZnLi alloy with Li after further discharge (Fig. 6c). This fact is identified by the peak position, which showed lower energy than Zn foil, as shown in first derivative curves. The oxidation state of Mn before the first discharge cycle is close to 3+ (peak C in the first derivative plot at similar position as Mn in Mn_2_O_3_). As the Mn K-edge and its first derivative curves shown in Fig. 6d,e, the Mn K-edge shows a continuous shift to lower edge energy, that is, the electrons are transferred to Mn^3+^ during the first discharge cycle. During the first discharge, the intensity of the pre-edge peak increased and the peak C became weak. The pre-edge peak is associated with transition to empty Mn 3*d* states. The single pre-edge peak indicates that the Mn is still 3+, but due to the change of the spinel phase, the 1*s*→3*d* transition increases. During the charge process, pre-edge structure changed to double-peaked structure, which indicated the transition of Mn^3+^ to Mn^2+^. These results correspond well with the Operando XRD results shown in Supplementary Fig. 23. After the first discharge cycle, the oxidation state of Zn slightly increases during charge and decreases during discharge process, indicating the cycling process between ZnLi alloy and metallic Zn nanoparticles. Simultaneously, the oxidation state of Mn changes from +3 to +2 in the charge process and goes back to +3 at the end of the discharge process, as identified by the reversible shifting of the pre-edge peak positions. The operando XAS studies prove the good chemical stability of the 2D holey ZMO nanosheets during charge/discharge process, showing no unwanted impurities and minimal side reactions occurred in the charge/discharge process.

## Discussion

In this work, a general synthetic strategy is developed by employing graphene oxide as a sacrificial template to prepare various 2D holey TMO nanosheets, including mixed metal oxides ZnMn_2_O_4_, ZnCo_2_O_4_, NiCo_2_O_4_, CoFe_2_O_4_, and simple metal oxides, such as Fe_2_O_3_, Co_3_O_4_, and Mn_2_O_3_. As potential anode materials for both lithium and sodium ion storage, the as-obtained 2D holey MTMO nanosheets exhibit superior electrochemical properties including high reversible capacity, excellent rate capability and cycling stability. This work combined *in-situ* TEM, XRD, with *in-situ* XAS measurements, and revealed for the first time the mechanical properties and morphology evolution of the 2D holey MTMO nanosheets during electrochemical processes. Operando XRD and XAS results show that holey ZMO nanosheets deliver high capacity due to the formation of ZnLi alloy as well as the reversible transformation between Mn^2+^ and Mn^3+^. *In-situ* TEM characterizations show that these 2D holey ZMO nanosheets inherit the strong mechanical properties from graphene oxides, maintaining the holey morphology and displaying minimal structural changes during the lithiation/delithiation processes and even under press states. Owing to potential merits of 2D nanostructures, tunable porosity and inherently strong mechanical stability, these 2D holey nanosheets could be promising candidates for emerging applications in energy storage and conversion systems, and electrocatalysis. The proposed general strategy would open up a promising avenue for designing 2D nanostructures for versatile materials, especially those with intrinsically non-layered structures, for broad applications in nanoscience and nanotechnology.

## Methods

### Synthesis of graphene oxide (GO)

GO was prepared from purified natural graphite by a modified Hummers' method. Simply, 10 g of graphite powder was first added to 15 ml of concentrated H_2_SO_4_. Five grams of K_2_S_2_O_8_ and 5 g of P_2_O_5_ were then added slowly. The as-obtained mixed solution was heat-treated at 80 °C for 6 h. After cooling to room temperature, the resultant was carefully diluted with distilled water, filtered and washed on the filter until the rinse water pH became neutral. The product was dried in air at ambient temperature overnight. Then the preoxidized graphite was added to 230 ml of concentrated H_2_SO_4_ cooled in an ice-water bath. Thirty grams of KMnO_4_ was added very slowly into the mixture with stirring and cooling. All the operations were carried out very slowly in a fume hood. The as-obtained mixture was then magnetically stirred at 35 °C for 30 min followed by slowly adding 460 ml of distilled water. The temperature of system increased to 98 °C during this process and was kept for another 15 min. After adding 1.4 l of distilled water followed by 10 ml of 30% H_2_O_2_ solution, solid products were formed and the reaction was terminated. The solid product was separated by centrifugation, washed repeatedly with 5% HCl solution, and then dialysed for a week.

### Synthesis of 2D holey MTMO nanosheets

The 2D holey MTMO nanosheets were prepared via a facile two-step approach. In a typical synthesis of 2D holey ZMO nanosheets, GO suspension was first formed by dispersing 30 mg GO power in 75 ml of ethylene glycol (EG) under sonication. Metal precursors were formed by dissolving 0.5 mmol of zinc acetates (Zn(CH_3_COO)_2_·2H_2_O) and 1.0 mmol of manganese acetates (Mn(CH_3_COO)_2_·4H_2_O) in 25 ml of EG. The above two systems were then mixed together, and stirred at ambient temperature for at least 60 min to obtain a homogenous suspension. The obtained suspension was heated at 170 °C for 2 h in a reflux synthesis system. The mixture was then allowed to naturally cool down, and the products were obtained by centrifuging and washing with ethanol for several times. The product (donated as ZMO precursors/GO) was then dried in vacuum at 80 °C overnight. In the second step of synthesis, the 2D holey ZMO nanosheets can be obtained through calcination of the as-made ZMO precursors/GO at 400 °C in air for 120 min with ramp rate of 0.5 °C min^−1^. To prepare control ZMO sample, same amount of the chemicals and EG solvent were used for the reaction without any GO added as the typical synthesis mentioned above. The rGO was prepared via the first step of the synthesis without Zn(CH_3_COO)_2_·2H_2_O and Mn(CH_3_COO)_2_·4H_2_O. To evaluate the influence of calcination temperature, the as-made ZMO precursors/GO was annealed at 400, 500 and 600 °C in air for 120 min with same heating rate (0.5 °C min^−1^), donated as 2D holey ZMO-400, 2D holey ZMO-500, 2D holey ZMO-600, respectively. 2D holey ZCO nanosheets were prepared with Zn(CH_3_COO)_2_·2H_2_O and Co(CH_3_COO)_2_·2H_2_O in the presence of GO as the same method mentioned above. 2D holey NCO nanosheets were prepared by replacing Zn(CH_3_COO)_2_·2H_2_O and Mn(CH_3_COO)_2_·4H_2_O with Ni(CH_3_COO)_2_·4H_2_O and Co(CH_3_COO)_2_·4H_2_O as the same method mentioned above. 2D holey CFO nanosheets were prepared by replacing Zn(CH_3_COO)_2_·2H_2_O and Mn(CH_3_COO)_2_·4H_2_O with Co(NO_3_)_2_·6H_2_O and Fe(NO_3_)_3_·9H_2_O as the same method mentioned above.

### Characterization

Powder XRD patterns were collected on a Philips Vertical Scanning difractometer to identify the phase of the as-synthesized samples. Scanning electron microscope, STEM (Hitachi S5500) and TEM (JEOL 2010F) were used to characterize the morphology of the samples. The TG analysis was tested by a TGA/SDTA851e thermogravimetric analyser under an air atmosphere from 25 to 850 °C at a heating rate of 10 °C min^−1^. *In-situ* TEM (JEOL 3,010) was carried out using a Nanofactory holder capable of biasing. In the beginning, 2D holey ZnMn_2_O_4_ nanosheets were loaded to one gold tip by conductive glue, and metallic Li was attached to the other Tungsten tip. During the sample transfer into the TEM chamber, Li metal was exposed to air for about 30 s and was oxidized to Li_2_O on the surface, which acts as the solid electrolyte. After the samples were loaded in the TEM, a +3 V bias (on the Li/Li_2_O side) was applied to initiate the lithiation process. The mechanical press/release was carried out after ZnMn_2_O_4_ nanosheets is fully lithiated and the bias is off.

### Electrochemical measurements

The working electrodes were prepared by mixing active materials (2D holey ZMO nanosheets) and polyvinylidene difluoride at a weight ratio of 90:10, in *N*-methyl-2-pyrrolidinone. Then the slurries were coated onto a copper foil. The as-prepared electrodes were dried under vacuum at 110 °C for 10 h. The loading of active materials was ∼1.0 mg cm^−2^. After being sealed, the electrodes were assembled into coin cells (CR2032) in an argon-filled glovebox using Celgard 2320 as separator, 1 mol l^−1^ LiPF_6_ in ethylenecarbonate and diethylenecarbonate (1:1, v/v) as the electrolyte and Li metal as the counter electrode. The assembled coin cells were tested on an Arbin battery test system with a voltage range of 0.01∼3.0 V. For comparison, the control electrode, named as Control ZMO, is made of free ZMO nanoplates without holes. Another control electrode, named as ZMO+SP, is made of free ZMO nanoplates physically mixed with Super P carbon in the weight ratio of 75:25. The 2D holey NCO nanosheets based electrode for sodium ion batteries were prepared by mixing active materials (2D holey NCO nanosheets), superP, sodium carboxymethyl cellulose (CMC) at a weight ratio of 80:10:10, in water. The electrolyte used for sodium ion battery tests was 1 M NaClO_4_ dissolved in propylene carbonate with 2%fluoroethylene carbonate additive.

### Data availability

The data that support the findings of this study are available from the corresponding authors on request.

## Additional information

**How to cite this article:** Peng, L. *et al*. Holey two-dimensional transition metal oxide nanosheets for efficient energy storage. *Nat. Commun.* **8,** 15139 doi: 10.1038/ncomms15139 (2017).

**Publisher's note**: Springer Nature remains neutral with regard to jurisdictional claims in published maps and institutional affiliations.

## Supplementary Material

Supplementary Information Supplementary Figures, Supplementary Tables and Supplementary References
