# Supplementary material for: Holey two-dimensional transition metal oxide nanosheets for efficient energy storage
Source: Nat Commun. 2017 Apr 26;8:15139. doi: 10.1038/ncomms15139 (PMC5414078; doi:10.1038/ncomms15139)
Supplement: Supplementary Information — Supplementary Figures, Supplementary Tables and Supplementary References [file ncomms15139-s1.pdf]

Supplementary Figures

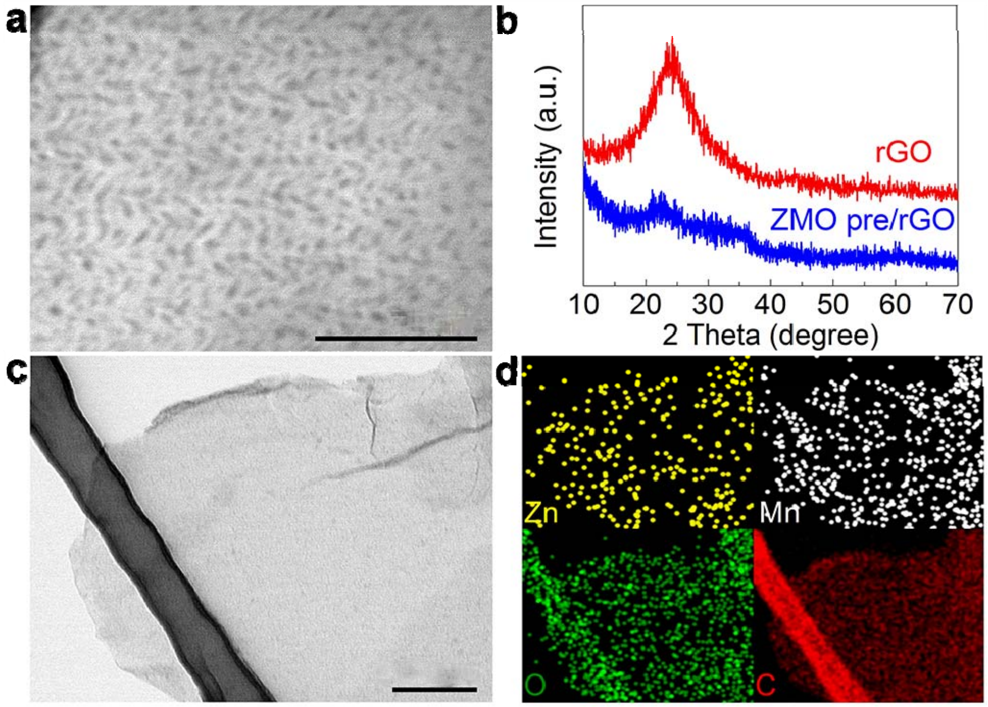

Supplementary Figure 1 | Characterization of ZMO precursor/rGO. (**a**) STEM image of ZMO precursor/rGO. Scale bar, 50 nm. (**b**) XRD patterns of reduced graphene oxide (rGO) and ZMO precursor/rGO. (**c**) Enlarged STEM image and (**d**) corresponding elemental mapping of ZMO precursor/rGO. Scale bar in **c**, 100 nm.

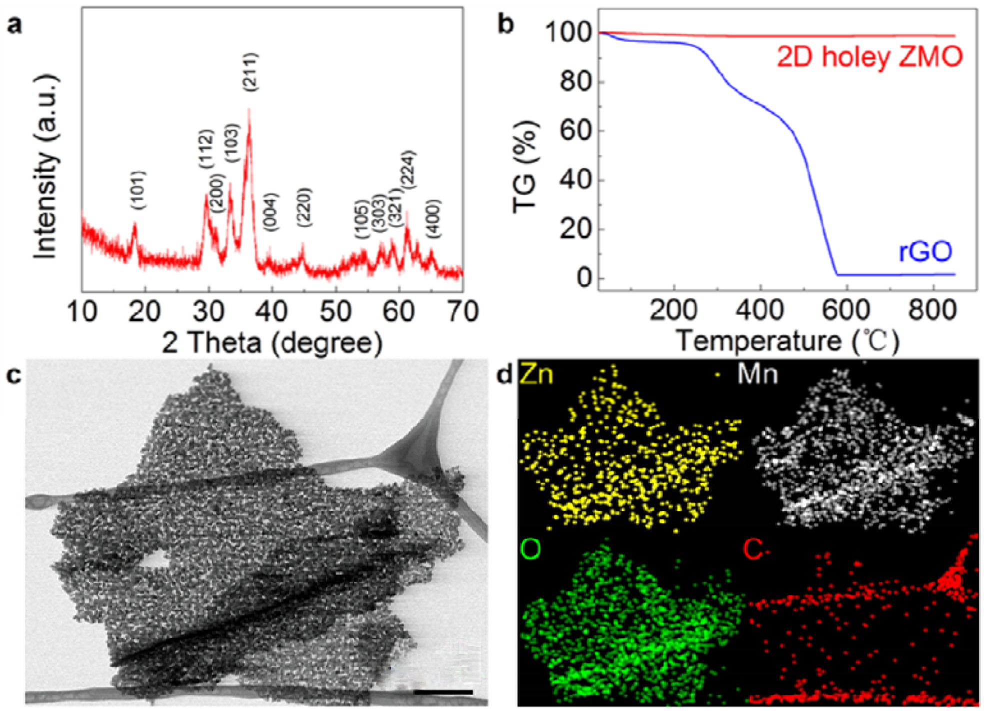

Supplementary Figure 2 | Characterization of 2D holey ZMO nanosheets. (**a**) XRD pattern of 2D holey ZMO nanosheets indicating the conversion of the precursor compound into spinel $ZnMn_2O_4$ (JCPDS card No. 24-1133). (**b**) TG analysis of reduced graphene oxide (rGO) and 2D holey ZMO nanosheets. (**c**) STEM image and (**d**) corresponding elemental mapping of 2D holey ZMO nanosheets. Scale bar in **c**, 200 nm.

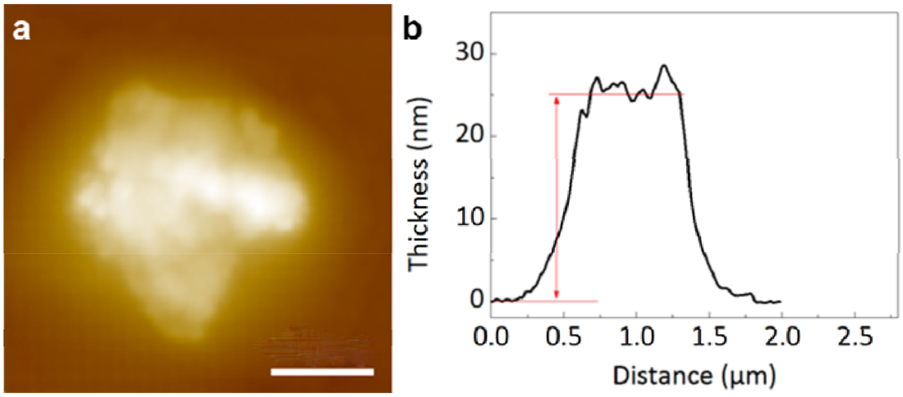

Supplementary Figure 3 | AFM image and the corresponding height profile of 2D holey ZMO nanosheets obtained under 400 ℃. (**a**) AFM image of ZMO nanosheets. Scale bar, 500 nm. (**b**) Height profile of ZMO nanosheets.

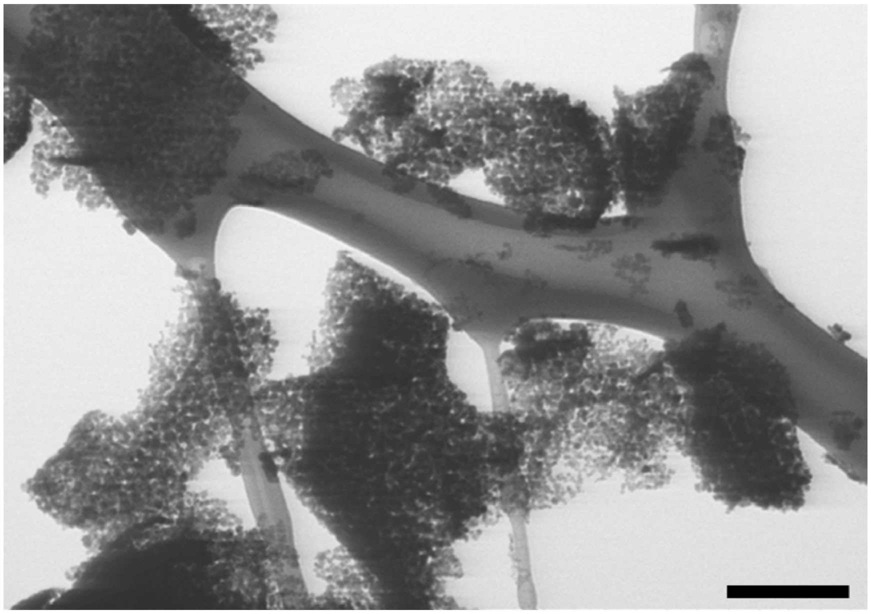

Supplementary Figure 4 | Low magnification SEM image of ZMO nanosheets obtained under 400℃. Scale bar, 200 nm.

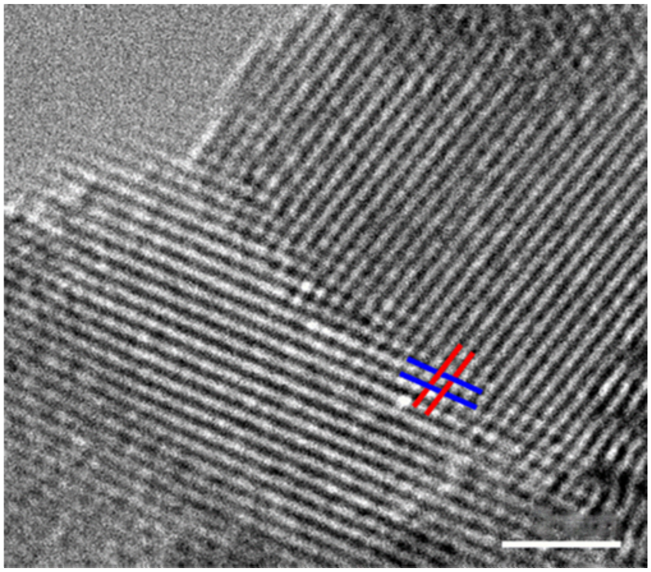

Supplementary Figure 5 | HRTEM image of 2D holey ZMO nanosheets. The crossed fringes in the overlapped domains reveal that the ZMO nanoparticles are chemically connected with each other. Scale bar, 2 nm.

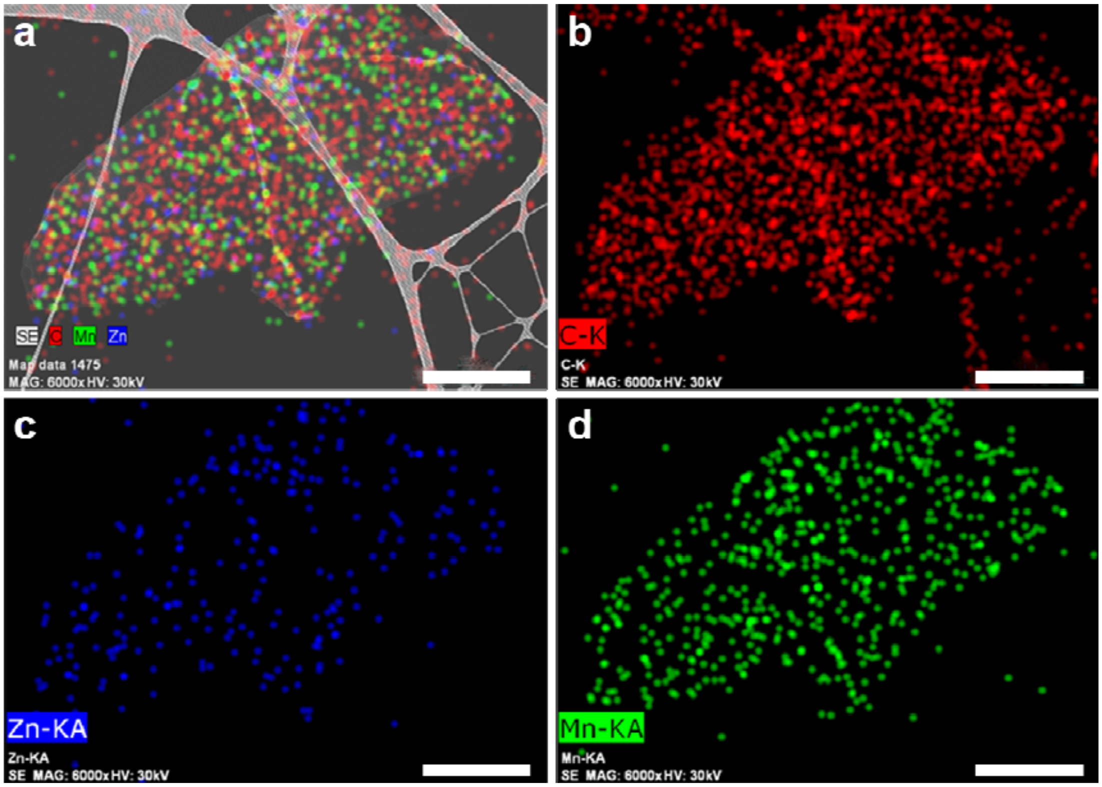

Supplementary Figure 6 | EDX mapping of GO nanosheets after stirring for enough time with Zn^{2+} and Mn^{2+} cations, showing the good adsorption of Zn^{2+} and Mn^{2+} cations onto GO nanosheets. (**a**) STEM image of GO nanosheets adsorbed with Zn^{2+} and Mn^{2+} cations. (**b**) C element distribution in the precursor nanosheets. (**c**) Zn element distribution in the precursor nanosheets. (**d**) Mn element distribution in the precursor nanosheets. Scale bar in all images, 5 μm.

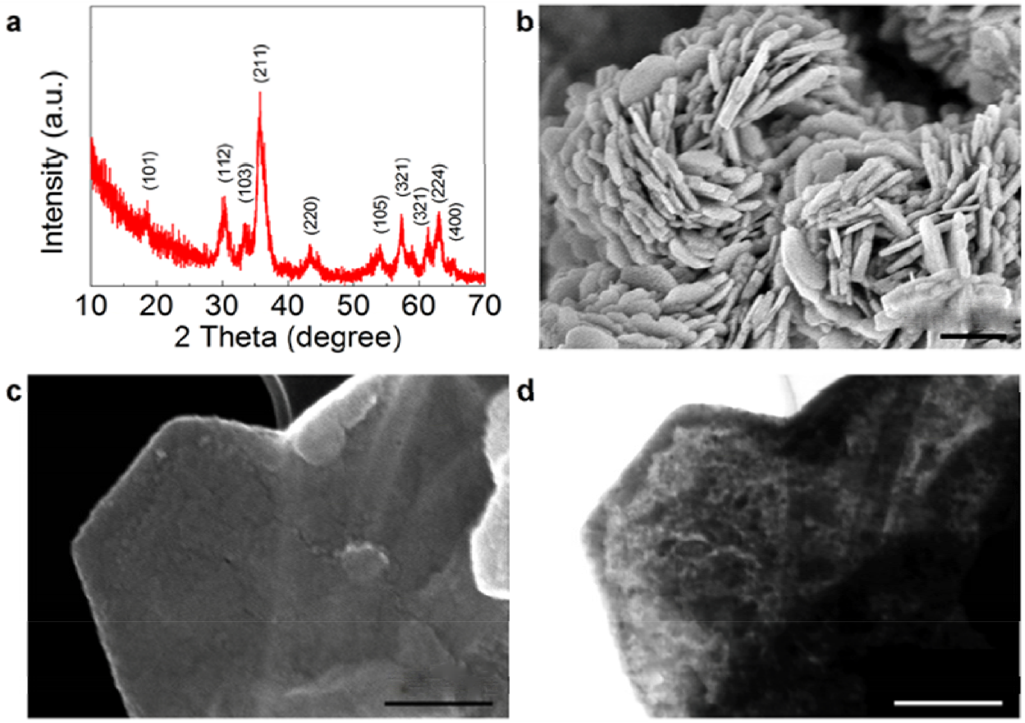

Supplementary Figure 7 | Characterization of free ZMO particles synthesized without addition of GO. (**a**) XRD patterns of free ZMO sample. (**b**) SEM image of free ZMO. Scale bar, 500 nm. (**c**) Enlarged SEM and (**d**) corresponding STEM images of free ZMO. Scale bar in **c** and **d**, 100 nm.

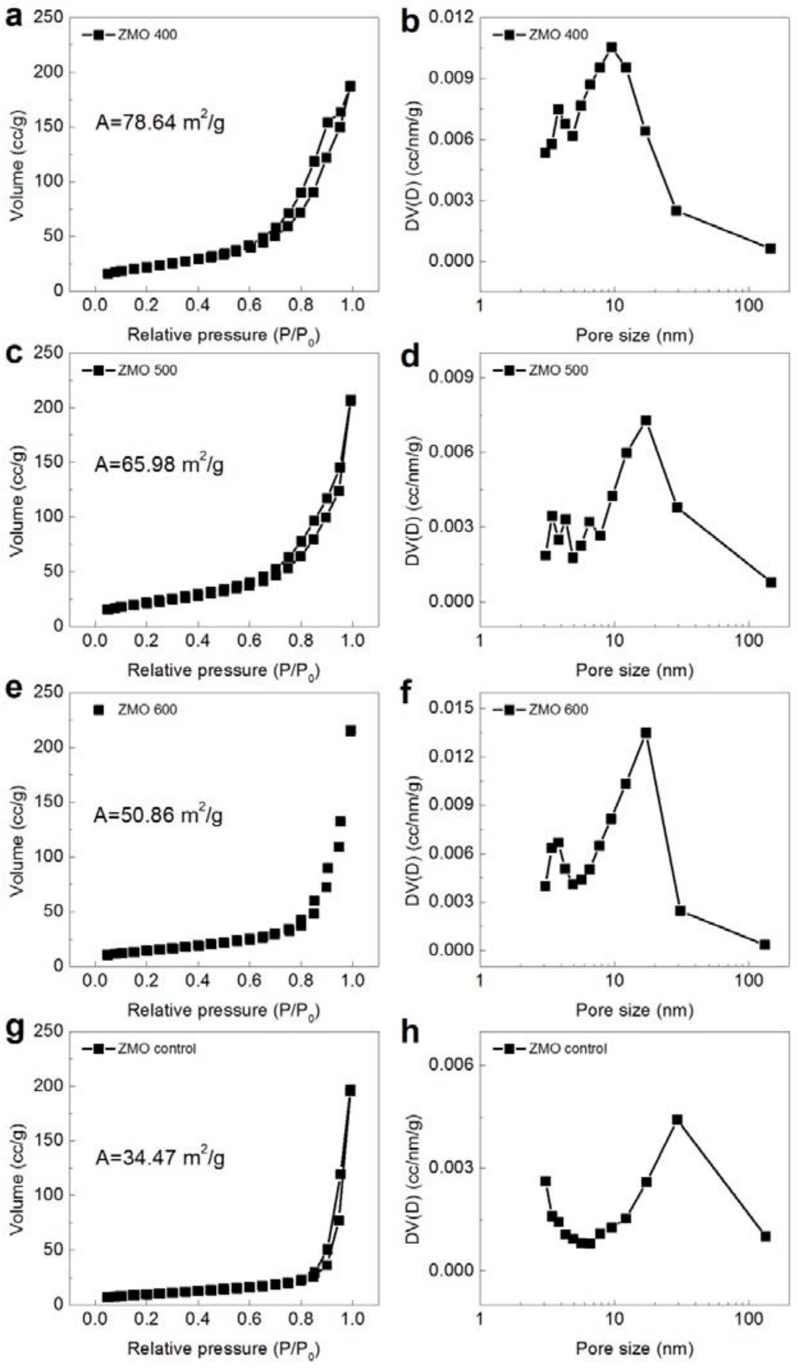

Supplementary Figure 8 | Isotherm results and BJH pore size distribution of ZMO nanosheets prepared at different calcination temperatures. (**a**, **b**) ZMO nanosheets at 400 °C; (**c**, **d**) ZMO nanosheets at 500 °C; (**e**, **f**) ZMO nanosheets at 600 °C; (**g**, **h**) control ZMO nanoplates.

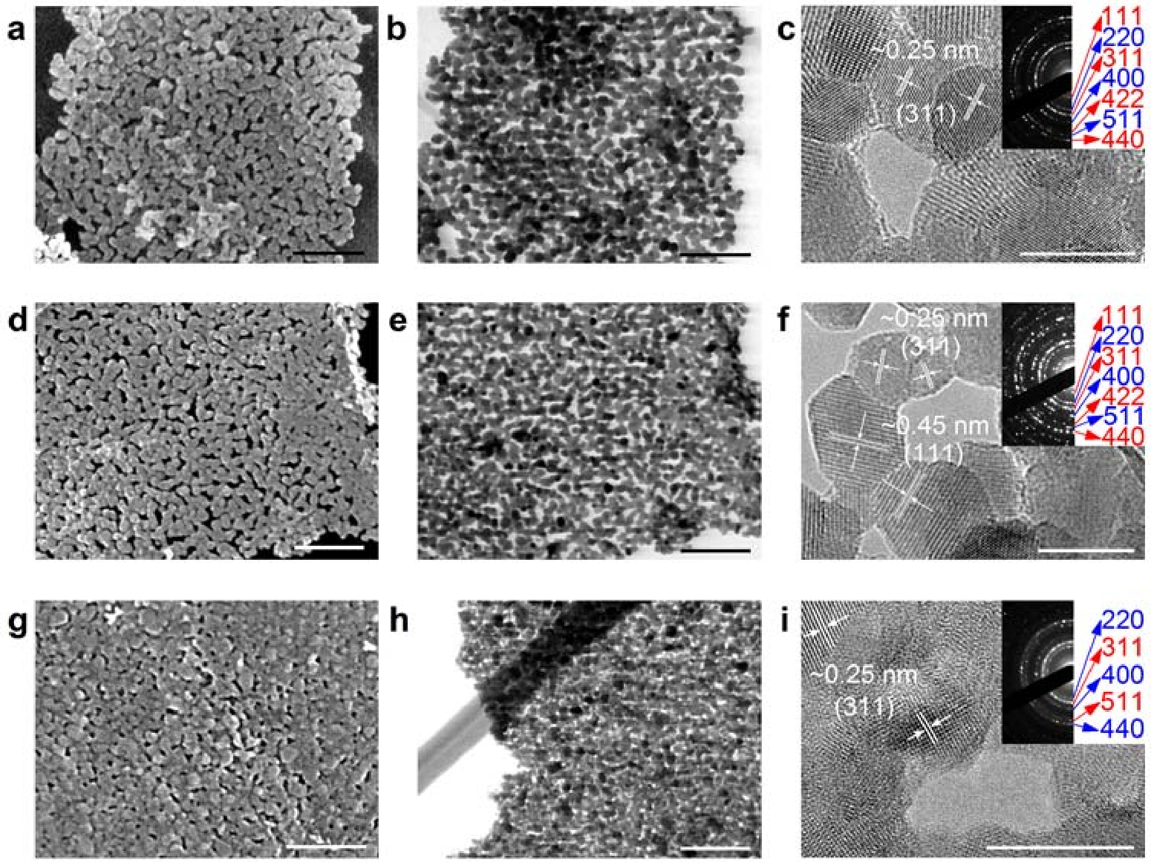

Supplementary Figure 9 | Electron microscopic images of versatile 2D holey MTMO nanosheets. SEM images (**a**, **d**, **g**), STEM images (**b**, **e**, **h**) and high-magnification TEM images and SAED patterns (**c**, **f**, **i**) of 2D holey nanosheets of ZCO (**a**-**c**), NCO (**d**-**f**), and CFO (**g**-**i**). Scale bar, 100 nm (**a**, **b**, **d**, **e**, **g**, **h**), 10 nm (**c**, **f**, **i**).

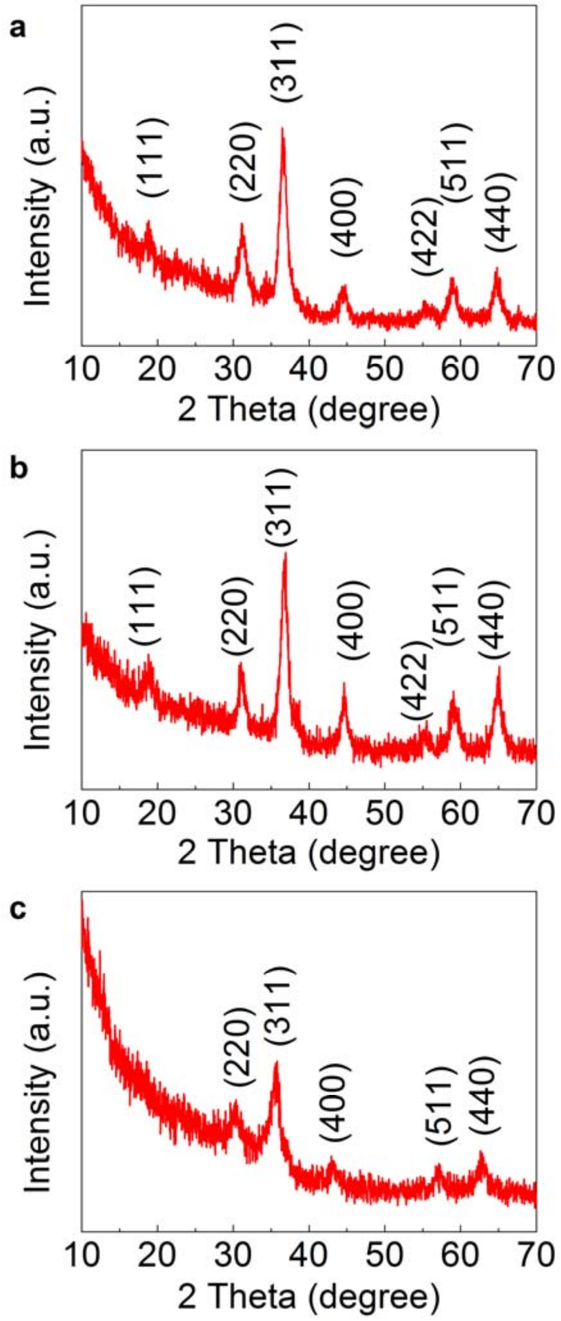

Supplementary Figure 10 | XRD patterns of 2D holey MTMO nanosheets. (**a**) 2D holey $ZnCo_2O_4$ (ZCO) nanosheets, (**b**) 2D holey $NiCo_2O_4$ (NCO) nanosheets, (**c**) 2D holey $CoFe_2O_4$ (CFO) nanosheets.

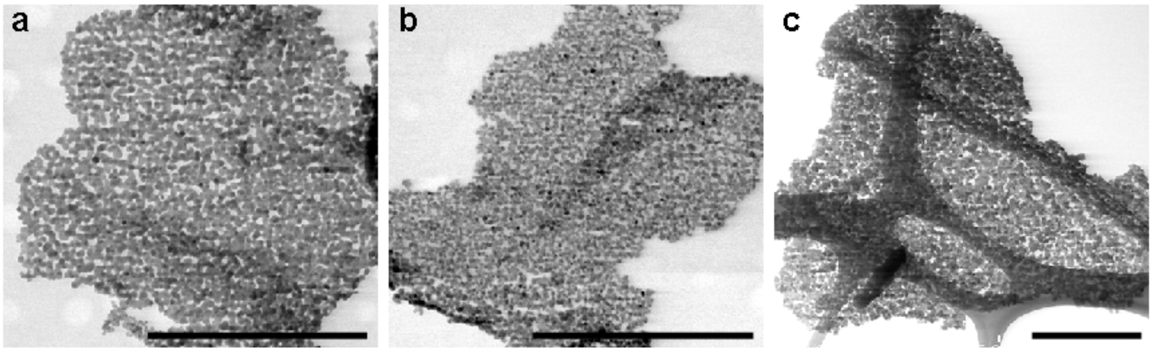

Supplementary Figure 11 | Scanning electron microscopic images of 2D holey simple TMO nanosheets. (**a**) 2D holey Co_3O_4 nanosheets; (**b**) 2D holey Mn_2O_3 nanosheets; (**c**) 2D holey Fe_2O_3 nanosheets. Scale bar in **a**, **b**, **c**: 500 nm.

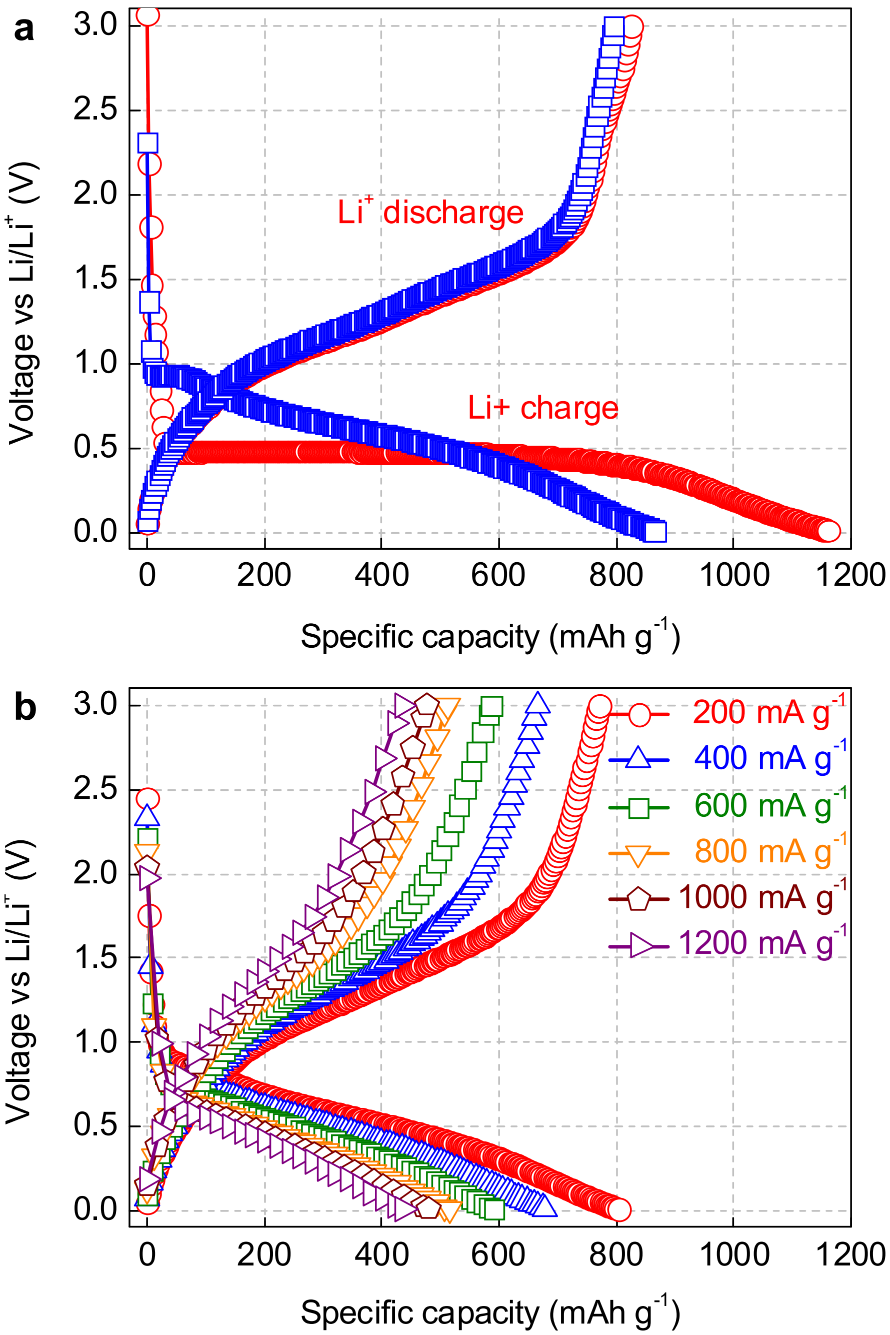

Supplementary Figure 12 | Electrochemical performances of 2D holey ZMO nanosheets. (**a**) Charge and discharge curves of 2D holey ZMO nanosheets for the first two cycles at a current density of 200 mA g^{-1}. (**b**) Representative charge and discharge curves of 2D holey ZMO nanosheets at various current densities.

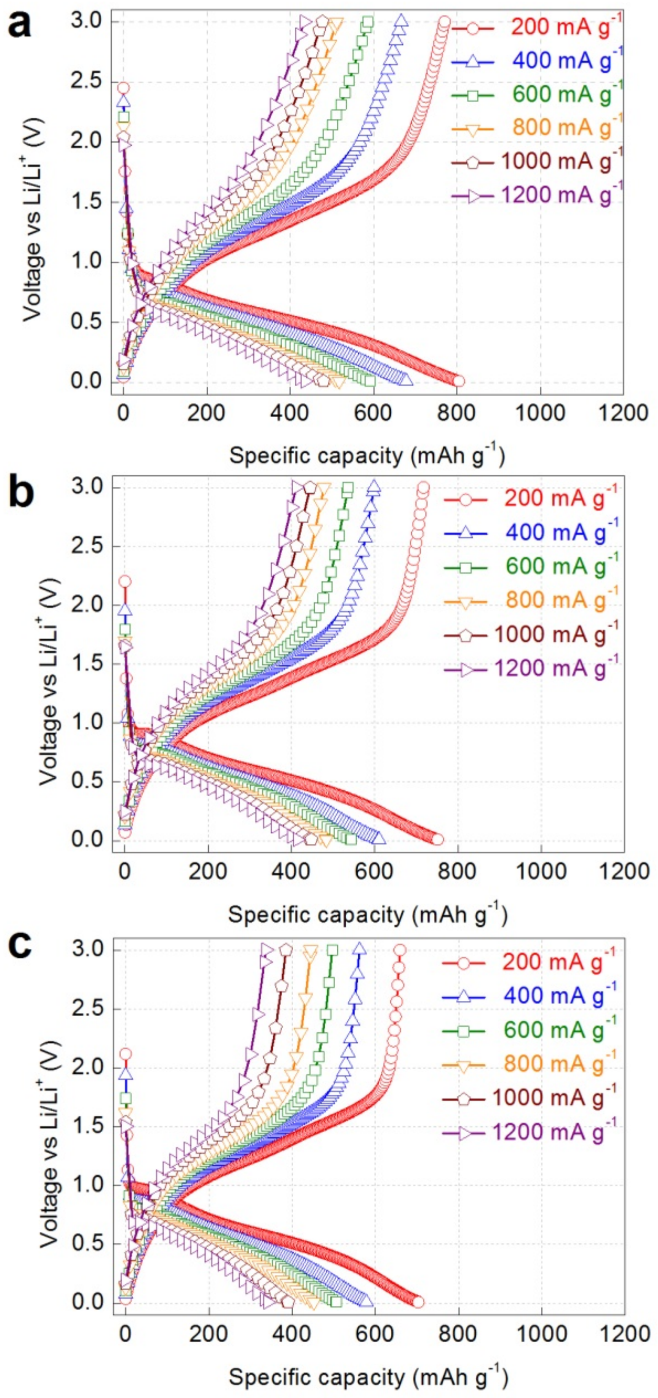

Supplementary Figure 13 | Charge/discharge curves of the holey ZMO nanosheets obtained at different temperatures at various current densities from 200 mA g^{-1} to 1200 mA g^{-1}. (**a**) 2D holey ZMO nanosheets obtained at 400 °C; (**b**) 2D holey ZMO nanosheets obtained at 500 °C; (**c**) 2D holey ZMO nanosheets obtained at 600 °C

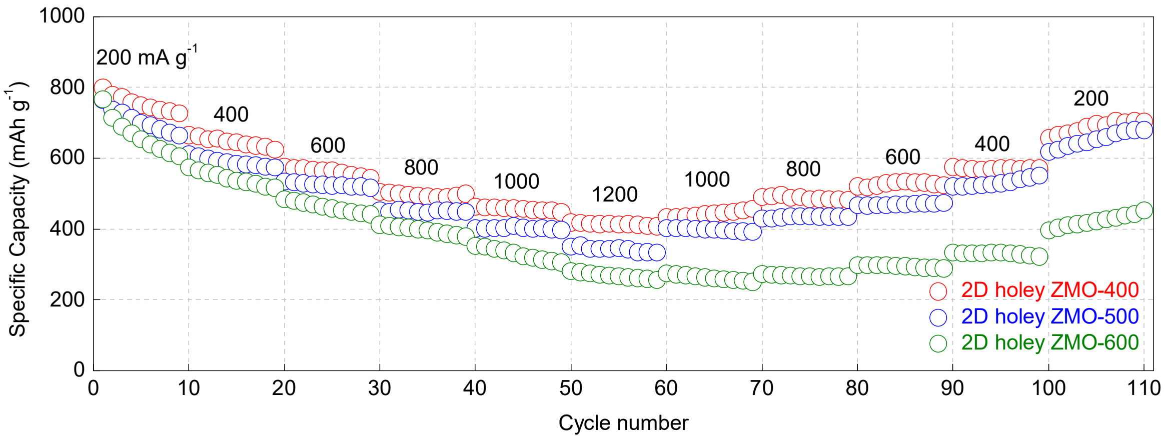

Supplementary Figure 14 | Rate performances of 2D holey ZMO nanosheets prepared at different temperature. 2D holey ZMO-400, 2D holey ZMO-500, and 2D holey ZMO-600 stand for the 2D holey ZMO nanosheets prepared at 400, 500, and 600 °C, respectively.

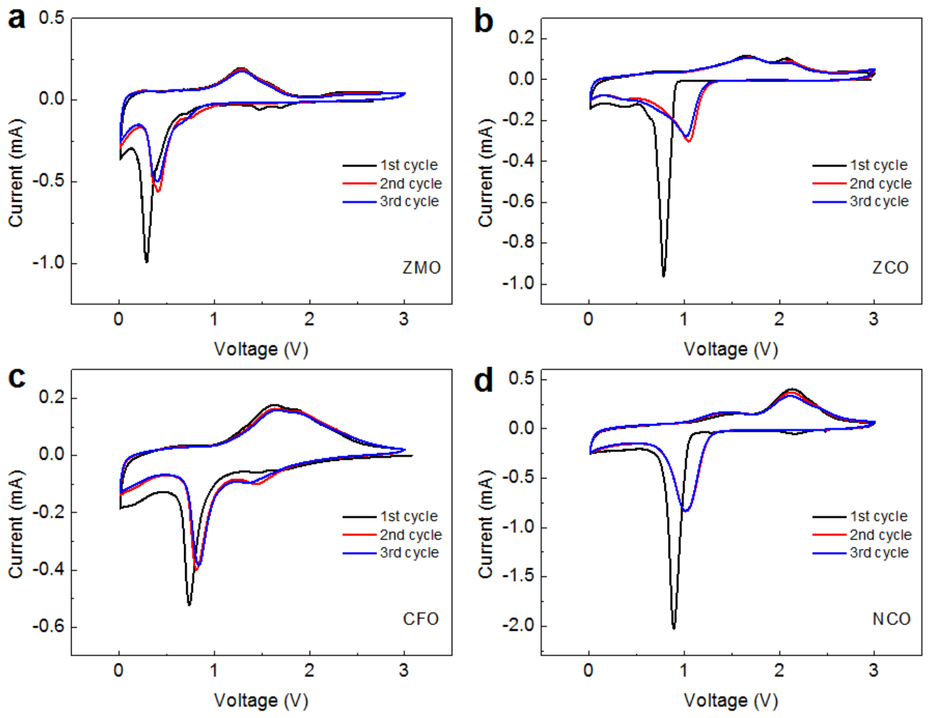

Supplementary Figure 15 | CV curves of the holey ZMO (a), holey ZCO (b), holey CFO (c) and holey NCO (d) nanosheets at scanning rate of 0.1 mV/s.

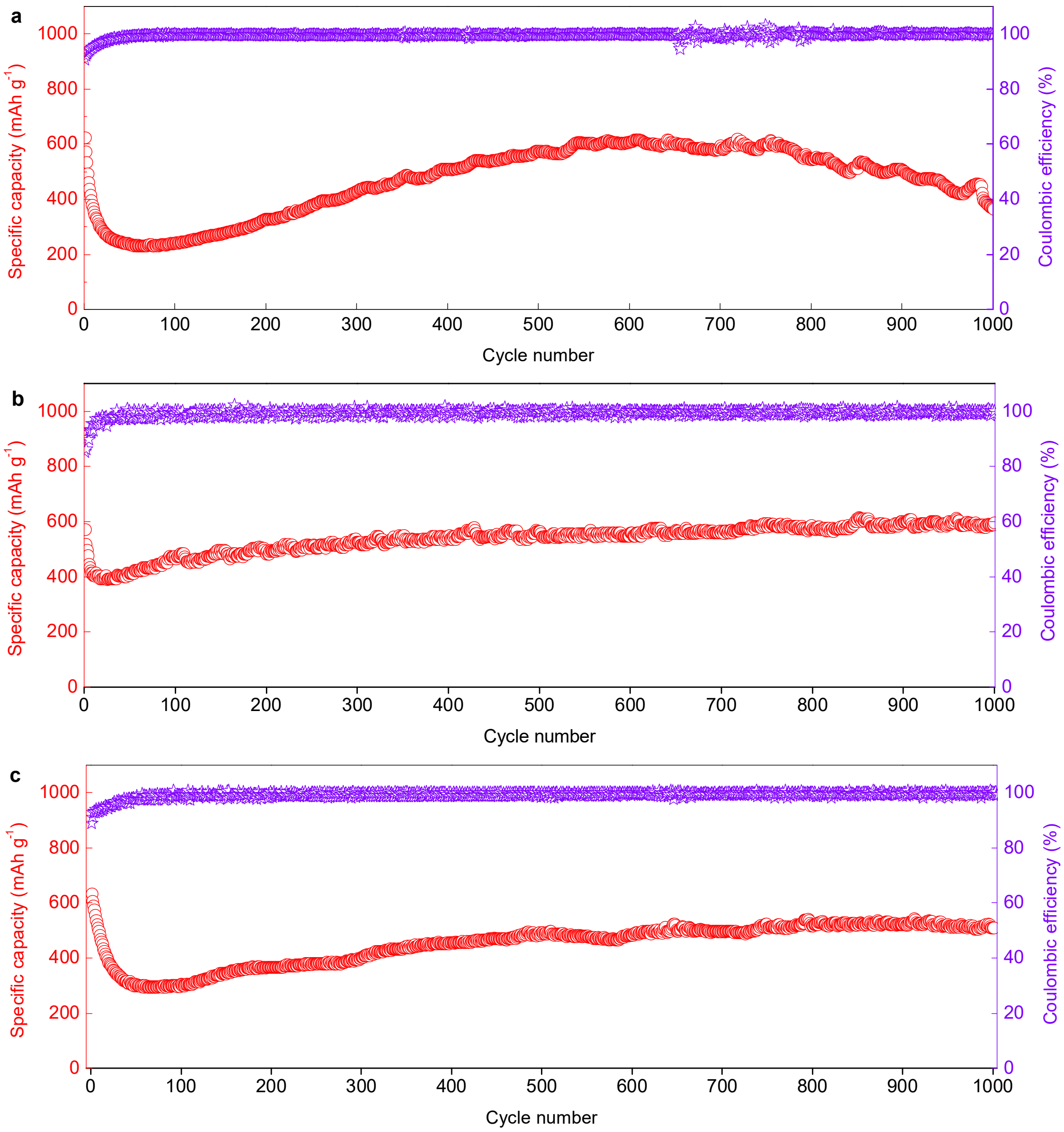

Supplementary Figure 16 | Cycling performance of 2D holey MTMO nanosheets as anodes for lithium-ion battery. (**a**) 2D holey ZCO nanosheets, (**b**) 2D holey NCO nanosheets (more reasonable data will get if possible), and (**c**) 2D holey CFO nanosheets at 1000 mA g^{-1} for 1000 cycles.

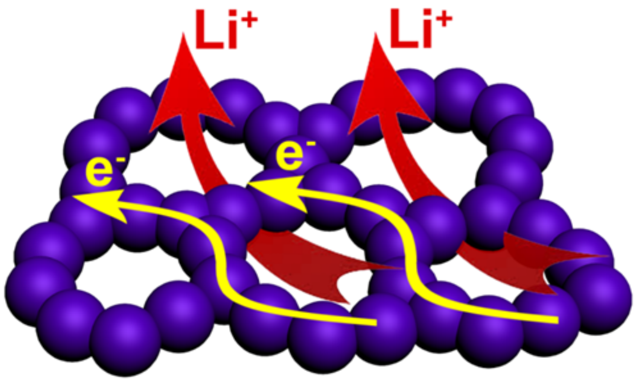

Supplementary Figure 17 | Schematic illustration of the electrochemical process. 2D holey MTMO nanosheets enable facile diffusion of Li^+ ion through the nano-holes, and continuous transport of electrons along within well interconnected nanocrystals.

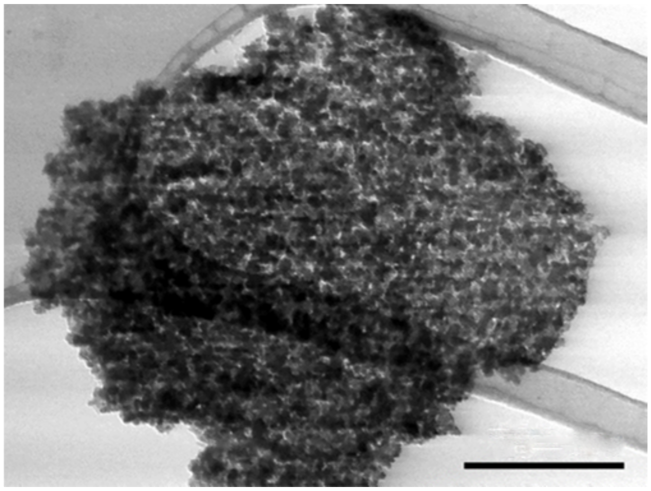

Supplementary Figure 18 | STEM image of 2D holey ZMO nanosheets after 100 cycles. The 2D holey nanostructures were reserved. To prepare the STEM sample, the cell was opened after several cycling, the 2D holey ZMO nanosheets anode washed with dimethyl carbonate (DMC) and sonicated in ethanol, and then the ethanol suspension was drop dried onto a STEM grid. Scale bar, 200 nm.

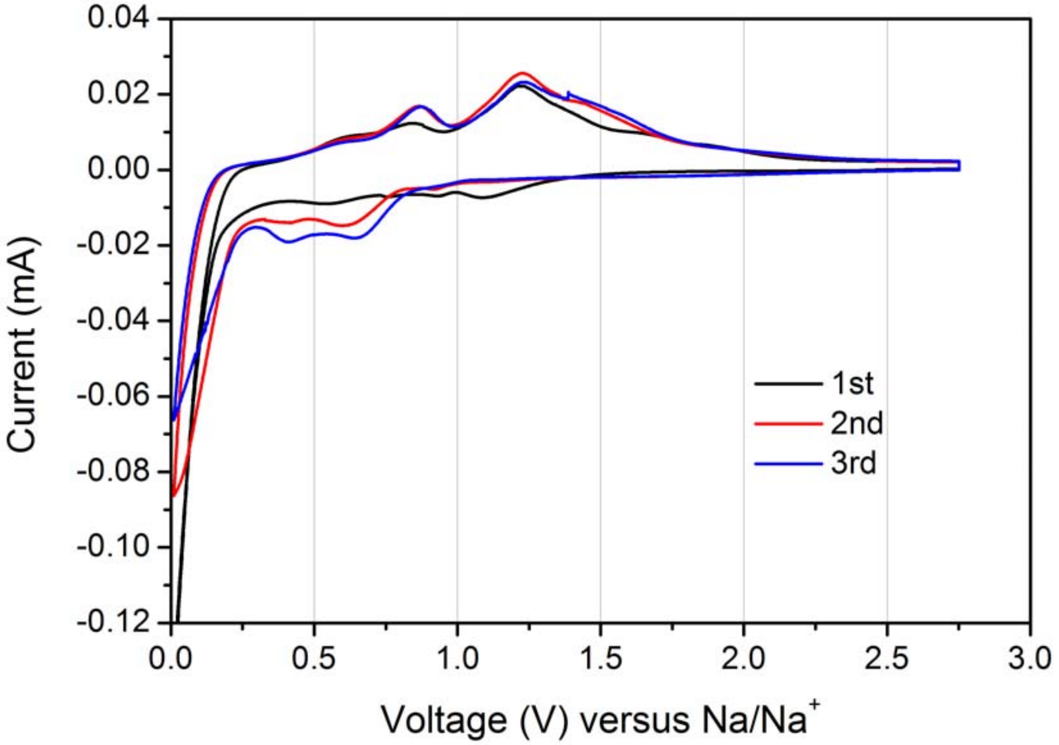

Supplementary Figure 19 | CV curves of the 2D holey NCO nanosheets for the first three cycles at the scan rate of 0.1 mV s^{-1}.

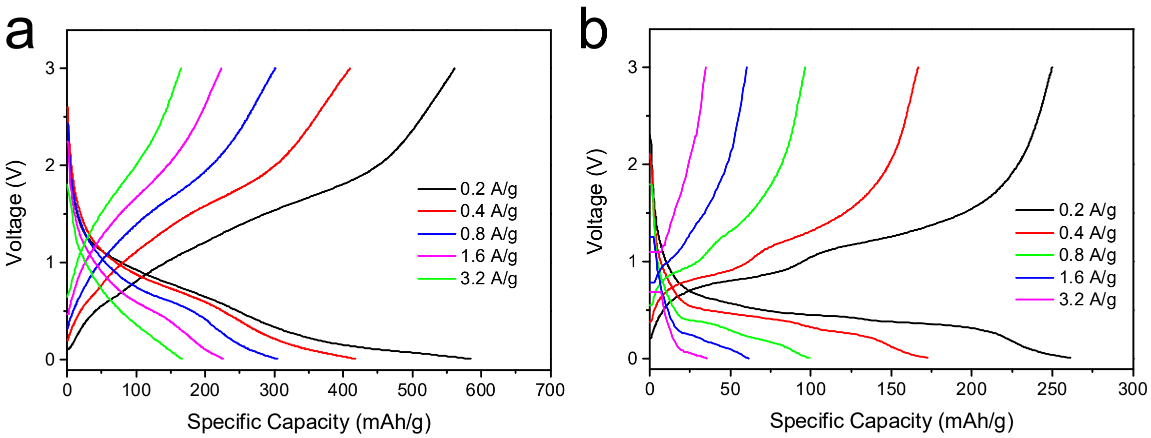

Supplementary Figure 20 | Charge-discharge curves of NCO electrodes at different current densities. (**a**) 2D holey NCO nanosheets; (**b**) controlled 2D NCO nanosheets without porosity.

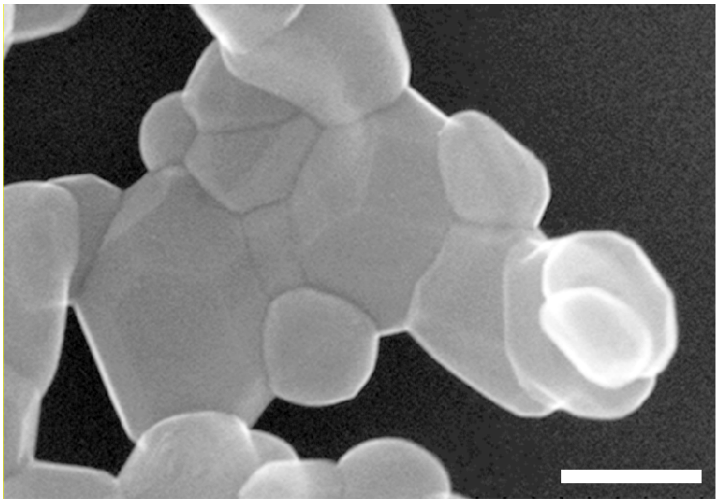

Supplementary Figure 21 | SEM image of the control 2D NCO nanoplates without porosity. Scale bar, 100 nm.

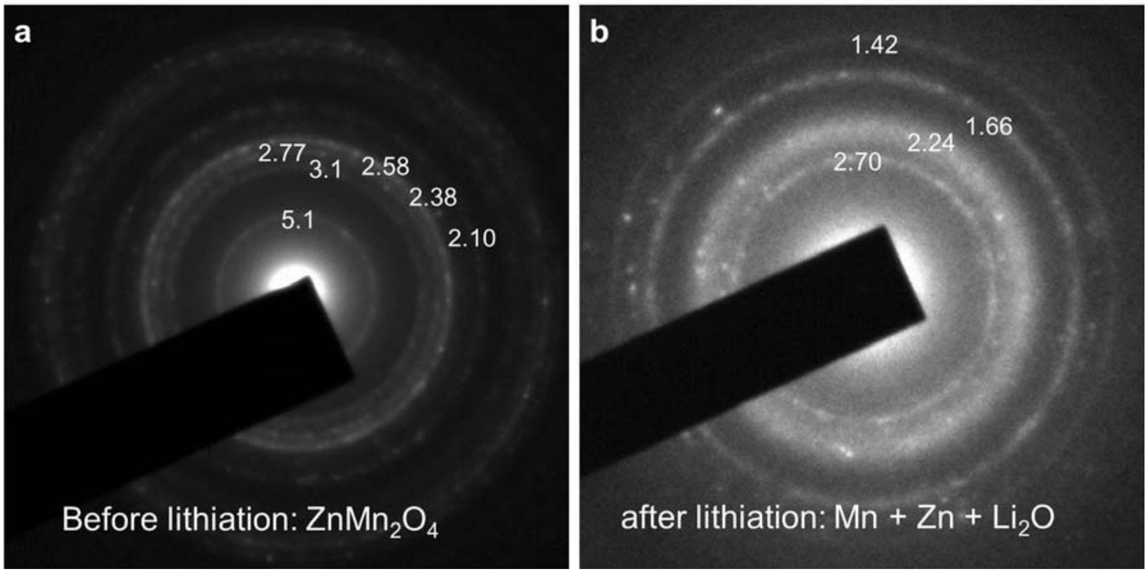

Supplementary Figure 22 | Selected area electron diffraction pattern of the ZMO nanosheets before and after lithiation. (**a**) Fresh ZMO nanosheets; (**b**) ZMO nanosheets after lithiation.

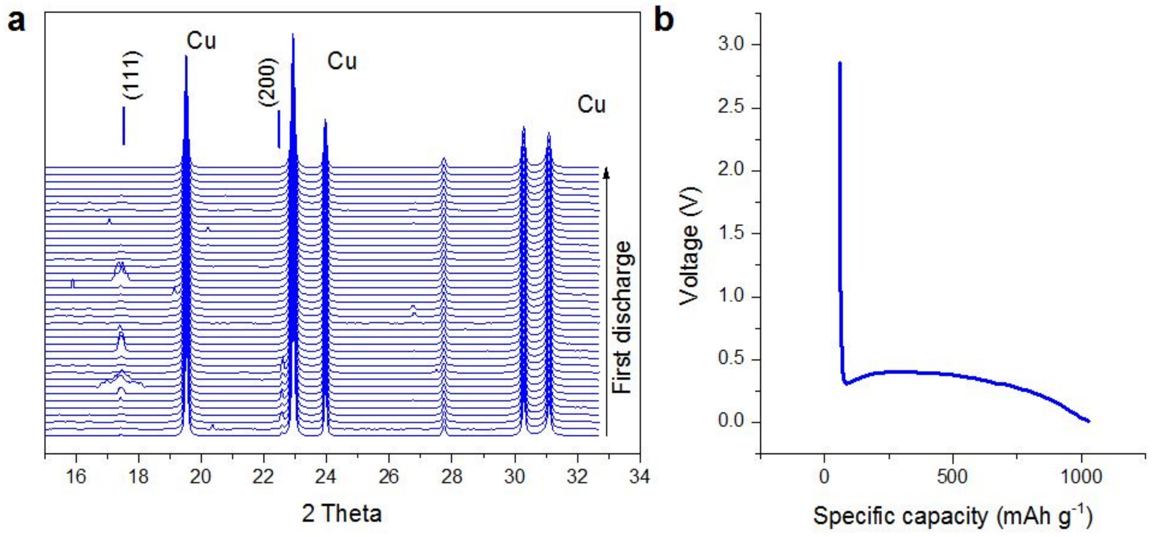

Supplementary Figure 23 | Operando XRD studies of 2D holey $ZnMn_2O_4$ nanosheets during the first discharge process. (**a**) XRD patterns of ZMO nanosheets during the first discharge process. (**b**) The discharge curve of the ZMO nanosheets. ZMO nanosheets deliver high capacity for Li ion storage via the conversion reaction along with Zn and Mn. A reversible capacity corresponding to 8.3 mol of Li per mole of $ZnMn_2O_4$ is expected, assuming x≤0.5 for Li intercalation into the spinel lattice ($ZnMn_2O_4 + xLi^+ + xe^- \rightarrow Li_x(ZnMn_2O_4)$), crystal structure destruction, followed by metal particle formation, and alloy formation with Zn ($Li_x(ZnMn_2O_4) + (8\text{-}x)Li^+ + (8\text{-}x)e^- \rightarrow Zn + 2Mn + 4Li_2O$; $Zn + Li^+ + e^- \leftrightarrow ZnLi$). These metallic nanoparticles are dispersed in a lithia (Li_2O) matrix, with the Li_2O + nanoparticles being surrounded by a solid electrolyte interface. This makes the mixtures amorphous, as identified by the operando XRD tests.

Supplementary Tables

Supplementary Table S1. A summary of recent studies on MTMO-based anode materials for lithium-ion battery.

**Active materials**	**Cycling stability ($mAh\ g^{-1}$)**	**Rate capability ($mAh\ g^{-1}$)**	**Ref**
ZMO nanoparticles		555 at 200 mA g^{-1}<br>280 at 2400 mA g^{-1}	*1*
ZMO nanowires	350 at 1000 mA g^{-1} (40 cycles)		*2*
ZMO nanorods	517 at 500 mA g^{-1} (100 cycles)	810 at 50 mA g^{-1}<br>457 at 1000 mA g^{-1}	*3*
ZMO tubules	784.3 at 100 mA g^{-1} (100 cycles)	644.6 at 100 mA g^{-1}<br>243.5 at 3200 mA g^{-1}	*4*
flower-like ZMO microstructures	626 at 100 mA g^{-1} (50 cycles)		*5*
ZMO hollow microspheres	607 at 400 mA g^{-1} (100 cycles)	791 at 200 mA g^{-1}<br>361 at 1600 mA g^{-1}	*6*
ZMO hollow microspheres	750 at 400 mA g^{-1} (120 cycles)	683 at 600 mA g^{-1}<br>396 at 1200 mA g^{-1}	*7*
ZCO nanoparticles	894 at 60 mA g^{-1} (60 cycles)		*8*
porous ZCO nanotubes	841 at 1000 mA g^{-1} (30 cycles)		*9*
porous ZCO nanoflakes	750 at 80 mA g^{-1} (50 cycles)		*10*
ZCO microsphere	721 at 100 mA g^{-1} (80 cycles)	970 at 100 mA g^{-1}<br>435 at 2000 mA g^{-1}	*11*
ZCO nanosheets	980 at 200 mA g^{-1} (200 cycles)	1034 at 200 mA g^{-1}<br>607 at 1000 mA g^{-1}	*12*
ZnO/ZCO nanosheets	730 at 500 mA g^{-1} (150 cycles)	880 at 200 mA g^{-1}<br>500 at 2000 mA g^{-1}	*13*

NCO nanocubes	1058 at 100 mA g^{-1} (100 cycles)		*14*
flower-like NCO microstructures	640 at 500 mA g^{-1} (60 cycles)	680 at 250 mA g^{-1}<br>420 at 2000 mA g^{-1}	*15*
NCO microsphere	705 at 800 mA g^{-1} (500 cycles)	1260 at 100 mA g^{-1}<br>393 at 1600 mA g^{-1}	*16*
NCO nanosheets	805 at 200 mA g^{-1} (100 cycles)	1018 at 200 mA g^{-1}<br>486 at 1000 mA g^{-1}	*17*
Mesoporous NCO nanosheets	204 at 200 mA g^{-1} (50 cycles)	142 at 1000 mA g^{-1}	*18*
NCO nanosheet networks	1170 at 200 mA g^{-1} (50 cycles)	1343 at 200 mA g^{-1}<br>780 at 2000 mA g^{-1}	*19*
NCO nanosheets	767 at 100 mA g^{-1} (50 cycles)	1149 at 100 mA g^{-1}<br>487 at 1000 mA g^{-1}	*20*
CFO microsphere	733.5 at 200 mA g^{-1} (50 cycles)	744.1 at 1000 mA g^{-1}<br>790.5 at 200 mA g^{-1}	*21*
ZMO nanosheets@CNT	625 at 306 mA g^{-1} (100 cycles)	819 at 62 mA g^{-1}<br>528 at 1224 mA g^{-1}	*22*
ZMO/graphene	707 at 100 mA g^{-1} (50 cycles)	980 at 100 mA g^{-1}<br>440 at 2000 mA g^{-1}	*23*
ZCO/Ni foam	1100 at 500 mA g^{-1} (50 cycles)		*24*
ZCO/Ni foam	900 at 416 mA g^{-1} (50 cycles)	1180 at 111 mA g^{-1}<br>485 at 1111 mA g^{-1}	*25*
ZCO/carbon fibers	1180 at 180 mA g^{-1} (100 cycles)		*26*
ZCO/carbon cloth	1200 at 200 mA g^{-1} (160 cycles)	1200 at 180 mA g^{-1}<br>605 at 4500 mA g^{-1}	*27*

ZCO nanosheets/graphene	961 at 90 mA g^{-1} (90 cycles)	963 at 90 mA g^{-1}<br>593 at 900 mA g^{-1}	*28*
NCO nanosheet/C	998 at 200 mA g^{-1} (50 cycles)	976 at 100 mA g^{-1}<br>603 at 800 mA g^{-1}	*29*
NCO/graphene	816 at 100 mA g^{-1} (70 cycles)	396 at 800 mA g^{-1}<br>974 at 100 mA g^{-1}	*30*
CFO/CNT	1045 at 200 mA g^{-1} (100 cycles)	1137.6 at 200 mA g^{-1}<br>621.7 at 2000 mA g^{-1}	*31*
CFO/graphene	565 at 800 mA g^{-1} (300 cycles)	1290 at 50 mA g^{-1}<br>730 at 800 mA g^{-1}	*32*
2D holey ZMO nanosheets	~430 at 800 mA g^{-1} (50 cycles)<br>~360 at 1000 mA g^{-1} (1000 cycles)	~770 at 200 mA g^{-1}<br>~430 at 1200 mA g^{-1}	*This work*
2D holey ZCO nanosheets	~360 at 1000 mA g^{-1} (1000 cycles)		*This work*
2D holey NCO nanosheets	~600 at 1000 mA g^{-1} (1000 cycles)		*This work*
2D holey CFO nanosheets	~510 at 1000 mA g^{-1} (1000 cycles)		*This work*

Supplementary References

1. Deng, Y. *et al.* Controllable synthesis of spinel nano-$ZnMn_2O_4$ via a single source precursor route and its high capacity retention as anode material for lithium ion batteries. *J. Mater. Chem.* **21**, 11987−11995 (2011).

2. Kim, S.-W. *et al.* Electrochemical performance and ex situ analysis of $ZnMn_2O_4$ nanowires as anode materials for lithium rechargeable batteries. *Nano Res.* **4**, 505−510 (2011).

3. Bai, Z. *et al.* Facile synthesis of loaf-like $ZnMn_2O_4$ nanorods and their excellent performance in Li-ion batteries. *Nanoscale* **5**, 2442−2447 (2013).

4. Kim, J. G., Lee, S. H., Kim, Y. & Kim, W. B. Fabrication of free-standing $ZnMn_2O_4$ mesoscale tubular arrays for lithium-ion anodes with highly reversible lithium storage properties. *ACS Appl. Mater. Interfaces* **5**, 11321−11328 (2013).

5. Xiao, L., Yang, Y., Yin, J., Li, Q. & Zhang, L. Low temperature synthesis of flower-like $ZnMn_2O_4$ superstructures with enhanced electrochemical lithium storage. *J. Power Sources* **194**, 1089−1093 (2009).

6. Zhou, L., Wu, H. B., Zhu, T. & Lou, X. W. Facile preparation of $ZnMn_2O_4$ hollow microspheres as high-capacity anodes for lithium-ion batteries. *J. Mater. Chem.* **22**, 827−829 (2012).

7. Zhang, G., Yu, L., Wu, H. B., Hoster, H. E. & Lou, X. W. Formation of $ZnMn_2O_4$ ball-in-ball hollow microspheres as a high-performance anode for lithium-ion batteries. *Adv. Mater.* **24**, 4609−4613 (2012).

8. Sharma, Y., Sharma, N., Subba Rao, G. V. & Chowdari, B. V. R. Nanophase $ZnCo_2O_4$ as a high performance anode material for Li-ion batteries. *Adv. Funct. Mater.* **17**, 2855−2861 (2007).

9. Luo, W., Hu, X., Sun, Y. & Huang, Y. Electrospun porous $ZnCo_2O_4$ nanotubes as a high-performance anode material for lithium-ion batteries. *J. Mater. Chem.* **22**, 8916−8921 (2012).

10. Qiu, Y., Yang, S., Deng, H., Jin, L. & Li, W. A novel nanostructured spinel $ZnCo_2O_4$ electrode material: morphology conserved transformation from a hexagonal shaped nanodisk precursor and application in lithium ion batteries. *J. Mater. Chem.* **20**, 4439−4444 (2010).

11. Hu, L. *et al.* Facile synthesis of uniform mesoporous $ZnCo_2O_4$ microspheres as a high-performance anode material for Li-ion batteries. *J. Mater. Chem. A* **1**, 5596−5602 (2013).

12. Zhu, Y. *et al.* Two-dimensional ultrathin $ZnCo_2O_4$ nanosheets: general formation and lithium storage application. *J. Mater. Chem. A* **3**, 9556-9564 (2015).

13. Zhen, M. *et al.* Synthesis of hierarchical $ZnO/ZnCo_2O_4$ nanosheets with mesostructures for lithium-ion anodes. *RSC Adv.* **6**, 43551-43555 (2016).

14. Guo, H. *et al.* Accurate hierarchical control of hollow crossed $NiCo_2O_4$ nanocubes for superior lithium storage. *Nanoscale* **6**, 5491−5497 (2014).

15. Li, L. *et al.* The facile synthesis of hierarchical porous flower-like $NiCo_2O_4$ with superior lithium storage properties. *J. Mater. Chem. A* **1**, 10935−10941 (2013).

16. Li, J., Xiong, S., Liu, Y., Ju, Z. & Qian, Y. High electrochemical performance of monodisperse $NiCo_2O_4$ mesoporous microspheres as an anode material for Li-ion batteries. *ACS Appl. Mater. Interfaces* **5**, 981−988 (2013).

17. Zhu, Y. *et al.* A Simple Synthesis of Two-Dimensional Ultrathin Nickel Cobaltite Nanosheets for Electrochemical Lithium Storage. *Electrochim. Acta* **176**, 141–148 (2015).

18. Zhou, K. *et al.* Mesoporous $NiCo_2O_4$ nanosheets with enhance sodium ion storage properties. *J. Alloys Compd.* **651**, 24–28 (2015).

19. Leng, X. *et al.* Ultrathin Mesoporous $NiCo_2O_4$ Nanosheet Networks as High-Performance Anodes for Lithium Storage. *ChemPlusChem* **80**, 1725–1731 (2015).

20. Mondal, A. *et al.* A Microwave Synthesis of Mesoporous $NiCo_2O_4$ Nanosheets as Electrode Materials for Lithium-Ion Batteries and Supercapacitors. *ChemPhysChem* **16**, 169–175 (2015).

21. Xiong, Q. Q. *et al.* Ascorbic acid-assisted synthesis of cobalt ferrite ($CoFe_2O_4$) hierarchical flower-like microspheres with enhanced lithium storage properties. *J. Power Sources* **256**, 153−159 (2014).

22. Yuan, C. *et al.* Heterostructured core–shell $ZnMn_2O_4$ nanosheets@carbon nanotubes' coaxial nanocables: a competitive anode towards high-performance Li-ion batteries. *Nanotechnology* **26**, 145401 (2015).

23. Zheng, Z., Cheng, Y., Yan, X., Wang, R. & Zhang, P. Enhanced electrochemical properties of graphene-wrapped $ZnMn_2O_4$ nanorods for lithium-ion batteries. *J. Mater. Chem. A* **2**, 149−154 (2014).
24. Qu, B. *et al.* High-performance lithium-ion battery anode by direct growth of hierarchical $ZnCo_2O_4$ nanostructures on current collectors. *ACS Appl. Mater. Interfaces* **6**, 731−736 (2013).
25. Long, H. *et al.* Synthesis of a nanowire self-assembled hierarchical $ZnCo_2O_4$ shell/Ni current collector core as binder-free anodes for high-performance Li-ion batteries. *J. Mater. Chem. A* **2**, 3741−3748 (2014).
26. Liu, B. *et al.* Advanced rechargeable lithium-ion batteries based on bendable $ZnCo_2O_4$-urchins-on-carbon-fibers electrodes. *Nano Res.* **6**, 525−534 (2013).
27. Liu, B. *et al.* Hierarchical three-dimensional $ZnCo_2O_4$ nanowire arrays/carbon cloth anodes for a novel class of high-performance flexible lithium-ion batteries. *Nano Lett.* **12**, 3005−3011 (2012).
28. Gao, G. *et al.* Growth of Ultrathin $ZnCo_2O_4$ Nanosheets on Reduced Graphene Oxide with Enhanced Lithium Storage Properties. *Adv. Sci.* **2**, 1400014 (2015).
29. Fan, Z. *et al.* A $NiCo_2O_4$ nanosheet-mesoporous carbon composite electrode for enhanced reversible lithium storage. *Carbon* **99**, 633–641 (2016).
30. Chen, Y. *et al.* Reduced graphene oxide networks as an effective buffer matrix to improve the electrode performance of porous $NiCo_2O_4$ nanoplates for lithium-ion batteries. *J. Mater. Chem. A* **2**, 4449−4456 (2014).
31. Zhang, Z. *et al.* Mesoporous $CoFe_2O_4$ nanospheres cross-linked by carbon nanotubes as high-performance anodes for lithium-ion batteries. *J. Mater. Chem. A* **1**, 7444−7450 (2013).
32. Liu, S. *et al.* Self-assembly of a $CoFe_2O_4$/graphene sandwich by a controllable and general route: towards a high-performance anode for Li-ion batteries. *J. Mater. Chem.* **22**, 19738−19743 (2012).
